# Vacuolar Sugar Transporter TMT2 Plays Crucial Roles in Germination and Seedling Development in Arabidopsis

**DOI:** 10.3390/ijms242115852

**Published:** 2023-11-01

**Authors:** Yanting Cao, Jinju Hu, Jinrong Hou, Chenguang Fu, Xingyue Zou, Xuxia Han, Pulian Jia, Chenjie Sun, Yan Xu, Yuhan Xue, Yiming Zou, Xinyue Liu, Xueying Chen, Guoyang Li, Jianing Guo, Min Xu, Aigen Fu

**Affiliations:** Chinese Education Ministry’s Key Laboratory of Western Resources and Modern Biotechnology, Key Laboratory of Biotechnology Shaanxi Province, Shaanxi Key Laboratory for Carbon Neutral Technology, Shaanxi Academy of Basic Sciences, College of Life Sciences, Northwest University, Xi’an 710069, China; caoyanting2017@163.com (Y.C.); jinjuhu@stumail.nwu.edu.cn (J.H.); houjinrong@stumail.nwu.edu.cn (J.H.); fuchenguang@stumail.nwu.edu.cn (C.F.); zouxingyue@stumail.nwu.edu.cn (X.Z.); 18835732860@163.com (X.H.); jiapulian@stumail.nwu.edu.cn (P.J.); sunchenjie@stumail.nwu.edu.cn (C.S.); xuyan000918@163.com (Y.X.); xueyuhan@stumail.nwu.edu.cn (Y.X.); zouyiming2020@163.com (Y.Z.); lxinyue202303@163.com (X.L.); chenxueying@stumail.nwu.edu.cn (X.C.); liguoyang@stumail.nwu.edu.cn (G.L.); jning0420@163.com (J.G.)

**Keywords:** tonoplast monosaccharide transporter 2 (TMT2), vacuole, sugar, seedling development, cotyledon

## Abstract

Vacuolar sugar transporters transport sugar across the tonoplast, are major players in maintaining sugar homeostasis, and therefore play vital roles in plant growth, development, and biomass yield. In this study, we analyzed the physiological roles of the tonoplast monosaccharide transporter 2 (TMT2) in Arabidopsis. In contrast to the wild type (WT) that produced uniform seedlings, the *tmt2* mutant produced three types of offspring: un-germinated seeds (UnG), seedlings that cannot form true leaves (*tmt2-S*), and seedlings that develop normally (*tmt2-L*). Sucrose, glucose, and fructose can substantially, but not completely, rescue the abnormal phenotypes of the *tmt2* mutant. Abnormal cotyledon development, arrested true leaf development, and abnormal development of shoot apical meristem (SAM) were observed in *tmt2-S* seedlings. Cotyledons from the WT and *tmt2-L* seedlings restored the growth of *tmt2-S* seedlings through micrografting. Moreover, exogenous sugar sustained normal growth of *tmt2-S* seedlings with cotyledon removed. Finally, we found that the TMT2 deficiency resulted in growth defects, most likely via changing auxin signaling, target of rapamycin (TOR) pathways, and cellular nutrients. This study unveiled the essential functions of TMT2 for seed germination and initial seedling development, ensuring cotyledon function and mobilizing sugars from cotyledons to seedlings. It also expanded the current knowledge on sugar metabolism and signaling. These findings have fundamental implications for enhancing plant biomass production or seed yield in future agriculture.

## 1. Introduction

In plant cells, up to 90% of the cell volume is occupied by the central vacuole, which is a single-layer membrane structure and the largest organelle. Based on different morphologies and functions, plant vacuoles can be classified into two types: lytic vacuoles (LVs) and protein storage vacuoles (PSVs) [1,2]. LVs are present in almost all vegetative tissues and functionally involved in substance storage, transport, and degradation. PSVs are mainly formed during the seed developmental stage and function as reservoirs for proteins, lipids, sugars, and inorganic substances [3]. LVs and PSVs are interchangeable; for example, LVs can turn into PSVs in maturing seeds, and PSVs can transform into LVs during germination [3]. Substance storage and utilization in vacuoles play pivotal roles in plant growth and development. In particular, seed germination is highly dependent on the utilization of stored substances in cotyledon vacuoles.

Seed germination, beginning with dry seed imbibition and ending with radicle protrusion, is the first stage of plant growth and is crucial for plant survival and reproduction. The germination process is affected by external environmental factors and internal seed factors. The former includes water, temperature, light, nitrate, oxygen, etc. The latter includes phytohormones, calcium ions, reactive oxygen species, and most importantly, storage compounds [4,5,6]. The major storage materials in seeds include oils, proteins, and carbohydrates. In Arabidopsis, oil is the major storage compound in seeds. However, oil stored in cotyledons does not directly move into the seedlings to support growth. Oil and high-molecular-weight carbohydrates need to be inverted to sugars and then further utilized by the seedlings [7].

Sugars provide energy to sustain plant life and also serve as essential signals to regulate plant growth and development. Sucrose, a major transportable form of photosynthetically assimilated carbon, is stored in vacuoles or transported from the source to the sink through the phloem [8,9,10]. Transporting sugars in and out of vacuoles is critical for plants to grow, develop, and respond to various environmental conditions [11,12]. Throughout their evolution, plants have developed numerous sugar transporters, channels, and pumps to transport sugars across the plant vacuolar membrane—the tonoplast. These carrier proteins are major players in maintaining sugar homeostasis in plant cells by storing sugar in the vacuoles when there is an excess and utilizing sugar when there is a need [13].

Plant sugar transporters are divided into three families: monosaccharide transporters (MSTs), sucrose transporters (SUC/SUTs), and sugars will eventually be exported transporters (SWEETs). MSTs and SUTs are members of the major facilitator superfamily (MFS), while SWEETs belong to the *Medicago truncatula* nodulation-induced gene 3 (*Mt*N3)/saliva family [14]. In Arabidopsis, more than 80 genes are thought to code for sugar transport proteins [15]. Up to now, 12 sugar transporters have been found to reside on the tonoplast in Arabidopsis, which include 3 vacuolar glucose transporters (VGTs), 3 tonoplast monosaccharide transporters (TMTs), also named TSTs, 3 SWEETs, sucrose transporter 4 (SUC4), early response to dehydration-like 6 (ERDL6), and early response to dehydration six-like 1 (ESL1) [8,16].

VGTs and TMTs belong to the MST (-like) gene family and mediate energy-dependent sugar transport driven by V-type H^+^-ATPases and vacuolar H^+^-PPases [8,16]. TMTs have been found to import sugars into the vacuoles in a proton antiport manner [17,18,19], and VGTs are likely to import glucose into the vacuoles in a similar way [20]. Among the three VGTs, only VGT1 (*At3g03090*) has been functionally characterized as a vacuolar importer, which transports glucose from the cytoplasm into the vacuoles and plays a vital role in seed germination and flowering [20]. As for the three *TMT* genes, *TMT1* and *TMT2* were well-expressed in different cell types and were induced to higher expression levels by drought, salt, cold, and sugar, while *TMT3* was barely expressed in Arabidopsis [19]. Under standard growth conditions, *TMT*-deficient mutants (single, double, and triple mutants) did not show visible phenotypic differences compared to the wild type (WT), but they contained reduced levels of glucose and fructose and a normal level of sucrose. When transferred to cold temperature conditions, WT plants accumulated higher levels of glucose, fructose, and sucrose; by contrast, *tmt1*, *tmt1*/*tmt2*, and *tmt1*/*tmt2*/*tmt3* plants accumulated less glucose and fructose, while *tmt1*/*tmt2* and *tmt1*/*tmt2*/*tmt3* contained a slightly reduced level of sucrose compared to the WT [19]. *TMT1* overexpression lines grew faster than WT plants, with increased seed biomass, lipid and protein content, higher *SUC2* expression, and increased sugar export rates. The *tmt1*/*tmt2* double mutant grew slower than the WT at 15 days after germination, but the growth difference became less pronounced after they had been growing for 34 days. Meanwhile, the *tmt1*/*tmt2* mutant plants produced 90% of 1000 seed weight and seed lipid and protein contents compared to the WT [17].

SWEETs were first identified as plasma membrane transporters responsible for transporting sucrose from the photosynthetic cells to the apoplast [21]. Later on, several SWEET members were found to be present on the tonoplast, facilitating sugar diffusion across the tonoplast [16]. We found that *SWEET2* is mainly expressed in Arabidopsis roots and acts as a vacuolar glucose importer, which plays key roles in transporting glucose into vacuoles, reducing carbon loss, and improving resistance to pathogens [22,23]. SWEET16 is a protein largely present in vascular parenchyma. It exports sugars from the vacuoles into the cytoplasm and further impacts seed germination and stress tolerance by maintaining sugar homeostasis in Arabidopsis [24]. Recently, SWEET16 has been found to be crucial for xylem development, as indicated by a high accumulation of SWEET16 at the procambium–xylem boundary and an abnormal xylem development in the *sweet16* mutant [25]. Different from SWEET2 and SWEET16, SWEET17 is a fructose-specific transporter and plays a key role in facilitating bi-directional fructose transport across the tonoplast and maintaining cytosolic fructose homeostasis [26]. SWEET17-dependent fructose released from the vacuoles plays a key role in regulating root growth during drought stress in Arabidopsis, as shown in the *sweet17* mutant, which exhibited reduced lateral root growth and impaired drought tolerance [27].

In contrast to TMT1/2 transporting glucose and sucrose into vacuoles, SUC4/SUT4 operates in the opposite direction, catalyzing sucrose export from the vacuoles [18]. SUC4 is exclusively localized to the tonoplast, and functions as a sucrose/H^+^ symporter for releasing sucrose from the vacuoles to the cytoplasm [28]. ERDL6, a vacuolar glucose exporter, is involved in maintaining cellular sugar homeostasis in Arabidopsis [29,30]. The expression of *AtERDL6* is highly induced by conditions that promote vacuolar glucose utilization, such as darkness, heat stress, and wounding, while it is down-regulated by conditions that trigger vacuolar glucose accumulation, such as cold stress and the external sugar supply. Interestingly, the *Aterdl6* mutant showed the phenotype of increased seed weight [29]. The overexpression of *Beta vulgaris* integral membrane protein (*Bv*IMP), the *At*ERDL6 homolog in sugar beet, resulted in impaired seed germination under conditions of sugar application or cold stress in Arabidopsis. Transgenic Arabidopsis plants with overexpressed *Bv*IMP accumulated fewer monosaccharides and exhibited reduced frost tolerance compared to the WT [30]. ESL1, another member of the ERD6-like family, is a low-affinity tonoplast monosaccharide facilitator that works in a proton-independent diffusion manner. Since *ESL1* showed a similar expression pattern to the vacuolar invertase, it was proposed that ESL1 might regulate osmotic pressure by mediating the cellular sugar content in coordination with the vacuolar invertase [31].

Over the past two decades, the identification and characterization of the vacuolar sugar carriers have expanded our knowledge of how those transporters direct sugar transport across the tonoplast and affect plant development. However, we think that many efforts to define the functions of vacuolar sugar transporters in plants are still inconclusive, especially in the process of germination and initial growth. Among the three Arabidopsis TMTs, only TMT1 has been extensively studied [17,19]. In an effort to explore the functions of Arabidopsis TMTs, we found that TMT2, a tonoplast sugar transporter that has not been well-characterized, plays a vital role in promoting germination and seedling development in Arabidopsis. The *tmt2* mutant showed impaired germination and arrested true leaf development. A small portion of *tmt2* plants, which have an elevated sugar content, grew as fine as the WT. The abnormal phenotypes of *tmt2* could be rescued by the application of sucrose, glucose, and fructose. RNA-seq data analysis showed that sugar shortage signaling stemmed from the deficiency of TMT2 changed global gene expressions, especially genes involved in energy metabolism, hormone signaling, and the target of rapamycin (TOR) pathway. We believe this research will shed light on the important roles of vacuolar sugar transporters in plant growth and development.

## 2. Results

### 2.1. Arabidopsis tmt2 Showed Impaired Germination and Arrested Seedling Growth

In Arabidopsis, vacuolar sugar transporters VGT1, TMT1, TMT2, TMT3, and SWEET2 were thought to import sugars from the cytoplasm to the vacuoles; moreover, SWEET2 was highly accumulated in roots, and the other four transporters were mainly expressed in aerial tissues [19,20,22]. RNA-seq and quantitative RT-PCR (qRT-PCR) experiments revealed that *VGT1*, *TMT1*, *TMT2*, and *TMT3* had different expression patterns in different tissues, and *TMT2* was well-expressed in the seedlings (Supplemental Figure S1A,B), suggesting that TMT2 could be a major player for vacuolar sugar transport in the early development process, and so it is worthy of further analysis.

**Figure 1:**
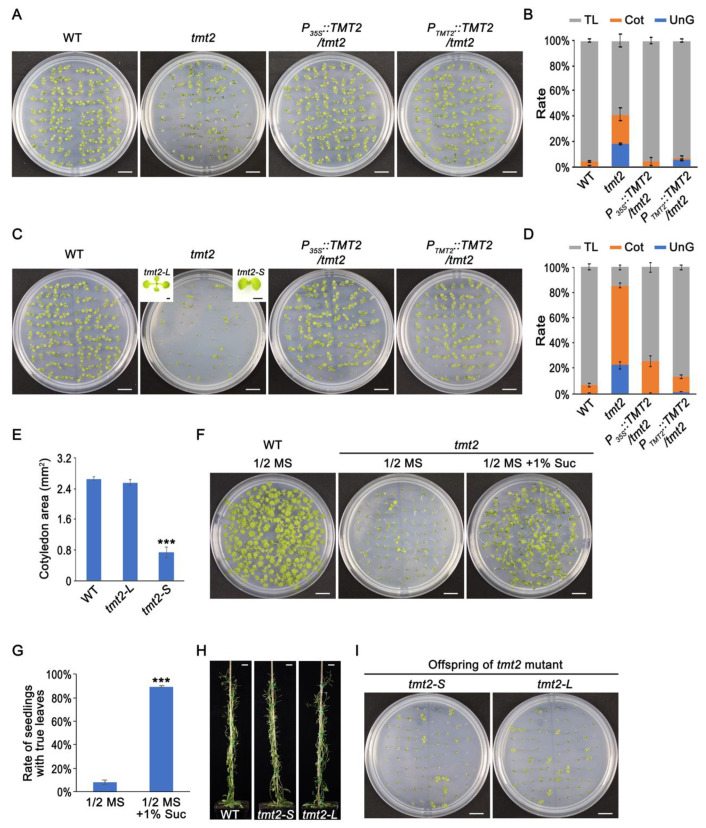
Phenotypes of *tmt2.* (**A**) Wild type (WT), *tmt2*, *P_35S_::TMT2*/*tmt2* and *P_TMT2_::TMT2*/*tmt2* complementation lines grown on 1/2 MS media with 1% sucrose for 7 days. (**B**) Rates of un-germinated seeds (UnG), seedlings arrested at the cotyledon stage (Cot) without true leaves, and true leaf stage seedlings (TL) of the WT, *tmt2*, *P_35S_::TMT2*/*tmt2*, and *P_TMT2_::TMT2*/*tmt2* on 1/2 MS media with 1% sucrose. (**C**) WT, *tmt2*, *P_35S_::TMT2*/*tmt2*, and *P_TMT2_::TMT2*/*tmt2* grown on 1/2 MS media for 7 days. Un-germinated *tmt2* seeds were designated as UnG, *tmt2* seedlings without true leaves were designated as *tmt2-S*, and *tmt2* seedlings grown normally were designated as *tmt2-L*. (**D**) Rates of UnG, Cot, and TL seedlings of the WT, *tmt2*, *P_35S_::TMT2*/*tmt2*, and *P_TMT2_::TMT2*/*tmt2* on 1/2 MS media. For (**A**,**C**), three repeats were performed. For (**B**,**D**), a total of 264 seeds of each sample were planted and growth was analyzed after 7 days. (**E**) Cotyledon area of 7-day-old WT, *tmt2-L*, and *tmt2-S* seedlings on 1/2 MS media. Sixty seedlings were analyzed. (**F**) Left: WT seedlings grown on 1/2 MS for 12 days. Middle: 5-day-old *tmt2-S* grown on 1/2 MS were transferred onto a new 1/2 MS media for a further 7 days of growth. Right: 5-day-old *tmt2-S* seedlings grown on 1/2 MS were transferred onto a new 1/2 MS media with 1% sucrose for a further 7 days of growth. (**G**) Statistics analysis of (**F**). A total of 264 seedlings of the WT and *tmt2* were transferred and growth was analyzed on the 7th day post-transfer. (**H**) Growth of WT, *tmt2-S* with additional 1% sucrose, and *tm2-L* plants after transferred into the soil for 6 weeks. (**I**) Offsprings of *tmt2-S* with additional 1% sucrose and *tmt2-L* plants grown on 1/2 MS media for 7 days in (**H**). Each experiment is a representative of three repeats. White scale bars = 1 cm, black scale bars = 1 mm. Error bars represent the SD of three biological replicates. Significant differences are indicated as ***, *p* < 0.001 by the Student’s *t*-test.

To analyze the function of TMT2 protein in the plant initial growth, we obtained a T-DNA insertion mutant line from NASC (Nottingham Arabidopsis Stock Center), SAIL_124_H03, which harbors two T-DNA insertions in its last exon (Supplemental Figure S1C, and also in Wormit et al., 2006) [19]. Only the transcript before the insertion site, not the full-length transcript of *TMT2*, could be detected by an RT-PCR analysis (Supplemental Figure S1D). It was found that *tmt1*, *tmt2*, and *tmt3* single mutants grew normally like the WT under standard growth conditions (on 1/2 MS agar media with 1% sucrose or in normal soil conditions) [19]. We first grew the *tmt2* mutant on 1/2 MS agar media with 1% sucrose and found that it grew similarly to the WT but with a lower germination rate (99.6% in WT vs. 82.2% in *tmt2*). We also found that more seedlings were arrested at the cotyledon stage without true leaves (23.1% Cot) compared to the WT (3.0% Cot) (Figure 1A,B).

Since it is a sugar transporter deficient mutant, we further grew *tmt2* on 1/2 MS media without sucrose. Again, *tmt2* showed a lower germination rate (77.7%) than the WT (99.6%), similar to plants grown on 1/2 MS media with 1% sucrose. Surprisingly, we found that a large portion of *tmt2* seedlings (63.3%) did not grow true leaves and could not develop further, which is in marked contrast to the fact that only a few WT seedlings (6.4%) could not produce true leaves (Figure 1C,D). For convenience, we designated un-germinated *tmt2* seeds as UnG, *tmt2* seedlings without true leaves as *tmt2-S*, and those with normal true leaves as *tmt2-L*. Comparing the growth of *tmt2* on 1/2 MS media without or with sucrose, we found that 1% sucrose only slightly improved the germination rate (77.7% without sucrose vs. 82.2% with sucrose), but substantially decreased the ratio of abnormal *tmt2-S* (63.3% without sucrose vs. 23.1% with sucrose) (Figure 1A–D). It is also noticeable that the cotyledons of *tmt2-S* were more dark-green and smaller than the cotyledons of the WT and *tmt2-L* (Figure 1A,C). We further measured the cotyledon sizes of the WT and *tmt2* and found that the cotyledon sizes of *tmt2-S* were much smaller than those of the WT, while the cotyledons of the WT and *tmt2-L* were almost the same size (Figure 1E). A more pronounced dark-green color and smaller size of the *tmt2-S* cotyledons implied abnormal development and malfunction of the *tmt2-S* cotyledons.

In order to test whether the 1% sucrose supply is the determinant factor for true leaf development, we transferred *tmt2-S* onto 1/2 MS media without sucrose and 1/2 MS media with 1% sucrose and recorded their subsequent growth. Not surprisingly, 84.1% of *tmt2-S* developed true leaves after growing on 1/2 MS media with 1% sucrose, and only about 8.3% could do so in the absence of sucrose (Figure 1F,G). This illustrated again that 1% sucrose was very efficient in helping abnormal *tmt2-S* develop into normal-like *tmt2-L*. To exclude other affecting factors in the media, we planted WT and *tmt2* seeds on 1/2 MS media with the coagulant phytagel media, and no obvious difference was found for plants grown on the phytagel media compared to those grown on the agar media (Supplemental Figure S1E).

Both *tmt2-L* and *tmt2-S* that were further grown with 1% sucrose were transferred into the soil, and they grew almost the same as the WT, normally producing seeds under the standard growth conditions (Figure 1H). The offspring of *tmt2-L* and sucrose-treated *tmt2-S* did not show visible differences when grown on 1/2 MS media without a sucrose supply, both producing a large amount of UnG and *tmt2-S* and a small amount of *tmt2-L* (Figure 1I). This demonstrated that sugar application could only complement the abnormal growth phenotypes of *tmt2* but could not cure its internal genetic *TMT2* deficiency. To confirm that the observed phenotypes of *tmt2* were caused by the deficiency of the *TMT2* gene, the full-length coding sequence of *TMT2* under the control of the *CaMV-35S* promoter and the native *TMT2* promoter were introduced into the *tmt2* mutant, respectively. The resulting transgenic plants were designated as *P_35S_::TMT2*/*tmt2* and *P_TMT2_::TMT2*/*tmt2*. RT-PCR and immunoblotting assays showed that the *TMT2* transgene was expressed well in *P_35S_::TMT2*/*tmt2* and *P_TMT2_::TMT2*/*tmt2* plants, which resembled the normal growth of the WT on 1/2 MS media with or without 1% sucrose, showing that the *TMT2* transgene was able to rescue the *tmt2* phenotypes (Figure 1A–D, Supplemental Figure S1F).

After analyzing the phenotypes of the plants grown on the media, we further examined the growth behavior of the plants grown in the soil. In order to make a qualitative comparison to previous studies [17,19], we included the *tmt1*/*tmt2* double mutant in this analysis. When planted in the soil, 0.8% of the WT, 19.4% of *tmt2*, 4% of *tmt1*/*tmt2*, 1.6% of *P_35S_::TMT2*/*tmt2*, and 3.2% of *P_TMT2_::TMT2*/*tmt2* failed to germinate, while 98% of the WT, 65.9% of *tmt2*, 85.7% of *tmt1*/*tmt2*, 93.7% of *P_35S_::TMT2*/*tmt2*, and 94% of *P_TMT2_::TMT2*/*tmt2* developed normal seedlings (Supplemental Figure S2). To our surprise, we found that *tmt2* plants grown in the soil were very similar to those grown on 1/2 MS media with 1% sucrose and not similar to those grown on 1/2 MS media only (Figure 1A–D and Supplemental Figure S2). Another interesting point is that the *tmt1*/*tmt2* double mutant behaved slightly better than the *tmt2* mutant but clearly worse than the WT, *P_35S_::TMT2*/*tmt2* and *P_TMT2_::TMT2*/*tmt2* lines (Supplemental Figure S2). This showed that *tmt1* is a suppressor of the *tmt2* mutation, implying that TMT1 and TMT2 may play antagonistic roles in early plant development.

In summary, the above analyses illustrated that the deficiency of *TMT2* caused abnormal phenotypes of the *tmt2* mutant with more UnG and *tmt2-S* offsprings, and TMT2 is a sugar transporter crucial for germination and initial seedling establishment.

### 2.2. Sugar Content of tmt2-L Was Elevated, While tmt2-S Maintained a WT-like Sugar Content

**Figure 2:**
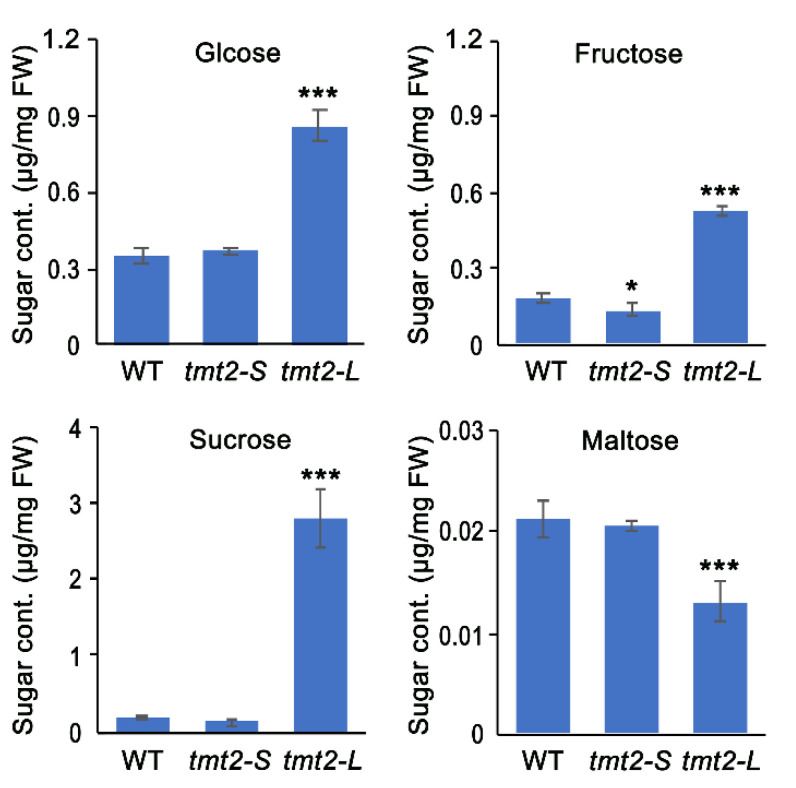
Glucose, fructose, sucrose, and maltose contents in 7-day-old WT, *tmt2-S*, and *tm2-L* seedlings. Error bars represent the SD of four biological replicates. Statistical analysis was performed by the Student’s *t*-test. * represents *p* < 0.05, *** represents *p* < 0.001.

Interestingly, *tmt2* showed three distinct growth phenotypes under the same genetic background: UnG failed to germinate, *tmt2-S* showed arrested true leaf development, and *tmt2-L* resembled the WT. We hypothesized that the cellular sugar content could be a determining factor for the striking discrepancy between *tmt2-S* and *tmt2-L* plants. In order to test this hypothesis, glucose, fructose, sucrose, and maltose contents in WT, *tmt2-S*, and *tmt2-L* seedlings that were grown on 1/2 MS media for 7 days were measured. The glucose, fructose, sucrose, and maltose contents in the WT were 0.349 ± 0.032 μg/mg, 0.178 ± 0.018 μg/mg, 0.186 ± 0.011 μg/mg, and 0.021 ± 0.002 μg/mg, respectively. Remarkably, the contents of glucose, fructose, sucrose, and maltose in *tmt2-S* were 0.373 ± 0.01 μg/mg, 0.143 ± 0.024 μg/mg, 0.141 ± 0.056 μg/mg, and 0.021 ± 0.001 μg/mg, respectively, which were almost the same as those in the WT. By contrast, the contents of glucose, fructose, sucrose, and maltose in *tmt2-L* seedlings were 0.86 ± 0.059 μg/mg, 0.53 ± 0.021 μg/mg, 2.787 ± 0.395 μg/mg, and 0.013 ± 0.002 μg/mg, respectively. The glucose and fructose contents in *tmt2-L* seedlings were about 2.5 times and 3 times higher than those in the WT and *tmt2-S*, whereas the sucrose content was 15 times higher than those in the WT and *tmt2-S*. Notably, the maltose content in *tmt2-L* was slightly lower (0.6-fold) than those in the WT and *tmt2-S* (Figure 2). In summary, the cellular sugar contents were significantly higher in *tmt2-L* while no obvious difference was observed in *tmt2-S* compared to the WT.

Regarding early-developed young seedlings, such as 7-day-old WT plants, the fresh weight of the cotyledons was 3 times higher than that of the true leaves (Supplemental Figure S3). Regarding the *tmt2-S* mutant, the sugar content came from the cotyledons since there was no true leaf development. Therefore, during the initial growth stage, the measured sugar content mainly reflected the cotyledon sugar content. It seems that sugar content in *tmt2* cotyledons similar to that in WT was not enough to promote its true leaf development. Only a significantly increased level of cotyledon sugar in *tmt2* was sufficient to sustain normal seedling development in the absence of TMT2.

### 2.3. Exogenous Sugar Significantly, but Not Completely, Rescued the Abnormalities of tmt2

**Figure 3:**
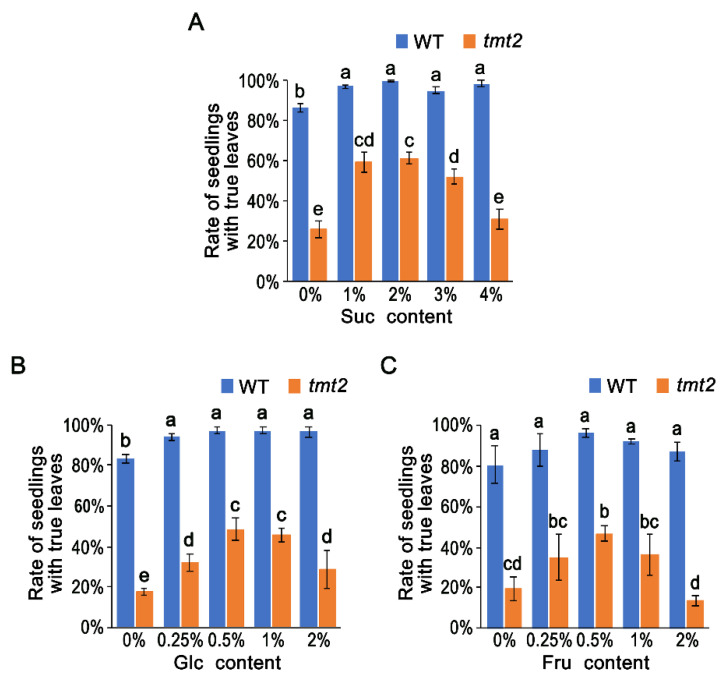
Sugar partially rescued the phenotypes of the *tmt2* mutant. Analysis of WT and *tmt2* seedlings with true leaves on 1/2 MS media with different contents of sugars: (**A**) sucrose, (**B**) glucose, (**C**) fructose. A total of 264 seeds of the WT and *tmt2* were planted and analyzed on the 7th day of growing. Error bars represent the SD of three biological replications. Different letters (a–e) indicate significant differences among samples: bc showed no significant difference with b or c, and cd showed no significant difference with c or d using one-way ANOVA with Tukey’s mean test (*p* < 0.05).

The growth and development of *tmt2* on 1/2 MS media with 1% sucrose was much better than without sucrose (Figure 1A–D), indicating that the insufficient carbon source in *tmt2* could be the major limiting factor. We postulated that an additional sugar supply could rescue the abnormal phenotypes of the *tmt2* mutant. In order to test this hypothesis, we fed WT and *tmt2* plants with sucrose at different concentrations on 1/2 MS media (Figure 3A). The growth and development of the WT did not change significantly under different concentrations of sucrose. By contrast, *tmt2* developed more *tmt2-L* plants when the sucrose content was increased to 2%. However, when the sucrose content was at 3% and 4%, the portion of *tmt2-L* plants decreased (Figure 3A and Supplemental Figure S4A). Sucrose with the optimum concentration (2%) could only promote about 60% of *tmt2* to develop into WT-like *tmt2-L* plants, indicating that sucrose could, but not completely, compensate for the deficiency of *TMT2*. Furthermore, *tmt2-L* seedlings were more sensitive to high concentrations of sucrose than the WT.

We also tested compensating the effects of glucose and fructose (Figure 3B,C, Supplemental Figure S4B,C). Similarly, WT plants showed almost the same growth with glucose and fructose at concentrations below 2%. The addition of glucose could also trigger *tmt2* to develop more *tmt2-L* plants but with a different dosage effect compared to sucrose. In detail, 0.5% glucose showed the best compensating effect on *tmt2*. A fructose supply at concentrations of 0.25% to 1% could lead *tmt2* to develop more *tmt2-L* plants, and 0.5% was the optimum concentration. However, the high concentration of fructose (above 2%) inhibited *tmt2* from developing true leaves, which was even worse than no fructose (Figure 3C). In summary, relatively low contents of sugar substantially rescued the abnormal phenotypes of *tmt2*, and higher contents of sugar inhibited the growth of *tmt2*. Compared to sucrose, both glucose and fructose showed more sensitive effects on rescuing the *tmt2* abnormal phenotypes.

In order to further explore the sensitivity of *tmt2* to higher contents of sugar, we grew the WT and *tmt2* seeds on 1/2 MS media with 1% sucrose. After 5 days of growth, WT and *tmt2-L* seedlings were moved to 1/2 MS media with 3% sucrose for an additional 7 days of growth, respectively. We found that 19.1% of the *tmt2-L* seedlings showed a phenotype of purple tissues, which is a typical stress response in Arabidopsis. By contrast, only 7.8% of WT seedlings on 1/2 MS media with 3% sucrose exhibited the purple color response. This result again demonstrated that the *tmt2* plants were significantly more sensitive to a high sugar content than the WT (Supplemental Figure S5).

### 2.4. Spatial Expression of TMT2 and Subcellular Localization of TMT2

**Figure 4:**
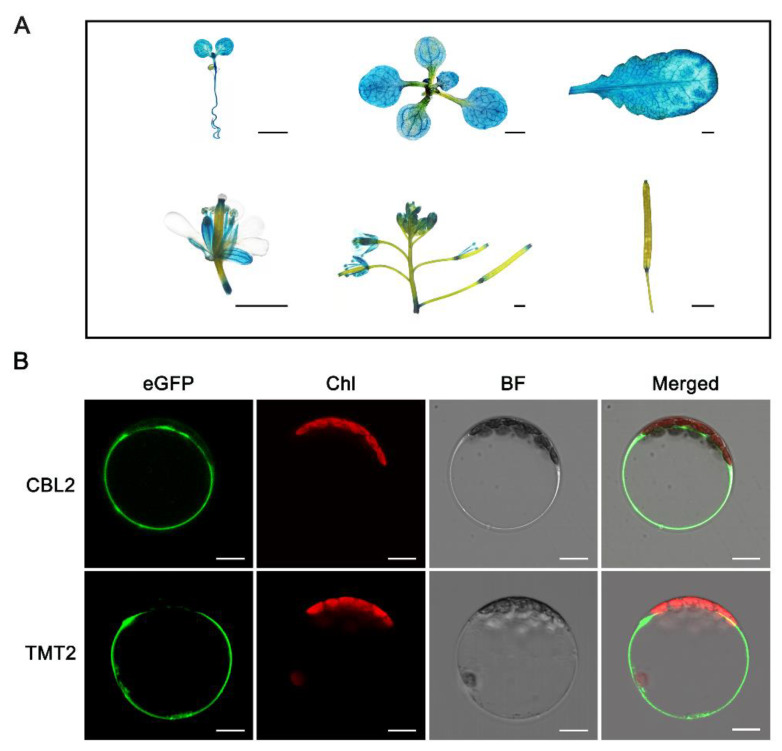
Expression pattern and subcellular localization of TMT2. (**A**) Histochemical GUS staining of the transgenic plants with the *P_TMT2_::GUS* gene. Scale bars = 2 mm. (**B**) Tonoplast localization of TMT2 in tobacco protoplast. CBL2 is a positive control located in the tonoplast. eGFP: enhanced green fluorescent protein, Chl: chloroplast, BF: bright field, GUS: beta-glucuronidase, CBL2: calcineurin B-like 2. Scale bars = 10 μm.

The *tmt2* mutant showed abnormalities in germination, cotyledon development, true leaf development, and response to high sugar stress, which led us to postulate that TMT2 might have a universal impact on the plant life. In order to test this hypothesis, we examined the spatial expression patterns of *TMT2* in Arabidopsis via a histochemical beta-glucuronidase (GUS) staining assay. The pCAMBIA1305*-P_TMT2_::GUS* construct, in which the *GUS* gene was driven by the native *TMT2* promoter, was transferred into WT plants, and the resulting GUS activity was recorded to indicate the spatial expression of *TMT2* (Figure 4A). It showed that the *TMT2* gene was actively transcribed in the cotyledons, roots, leaves, and other tissues in the vegetative growth stage. As for plants in the reproductive stage, *TMT2* expression mainly occurred in the petals, filaments, pollen cells, and silique nodes. The results from the GUS staining assay are very consistent with those from RNA-seq analysis and qPT-PCR analysis (Supplemental Figure S1A,B). It supported our conclusion that *TMT2* is actively involved in most physiological processes during the plant life cycle.

Germination and initial development are highly dependent on the normal function of the cotyledons. Severe phenotypes of *tmt2* implied that TMT2 should be a vital player in maintaining the normal cotyledon function. In order to explore the presence of the TMT2 protein in cotyledons, we examined the TMT2 protein level in the cotyledons of the *P_TMT2_::TMT2*/*tmt2* complementation line, and again, we found that the TMT2 protein was well-expressed in the cotyledons (Supplemental Figure S6).

The subcellular localization of a protein determines its function in a cell. In order to better understand the function of TMT2, we performed a subcellular localization assay with the well-known tonoplast calcineurin B-like 2 (CBL2) protein as the reference [32]. As expected, the fluorescence signal from CBL2-eGFP is found on the tonoplast of transgenic tobacco protoplasts, and the fluorescence signal from TMT2-eGFP showed the same pattern as that from CBL2-eGFP. We also noted that the autofluorescence from chlorophyll did not overlap with the fluorescence from TMT2-eGFP and CBL2-eGFP (Figure 4B). The same patterns shown by TMT2-eGFP and CBL2-eGFP indicated that the TMT2 protein resides on the tonoplast, but not on the plasma membrane, similar to CBL2.

### 2.5. Growth after the Initial Stage of tmt2-L Was Fundamentally Normal

Once the *tmt2* mutant developed into *tmt2-L*, it grew as well as the WT and normally produced seeds after further growth in the soil. In vivo chlorophyll fluorescence analysis showed that photosynthesis parameters of 24-day-old *tmt2-L*, including Fv’/Fm’, Y(PSII), 1-qP, and NPQ were almost identical to those of the WT, indicating no difference in the photosynthetic capacities between the WT and *tmt2-L* (Supplemental Figure S7).

**Figure 5:**
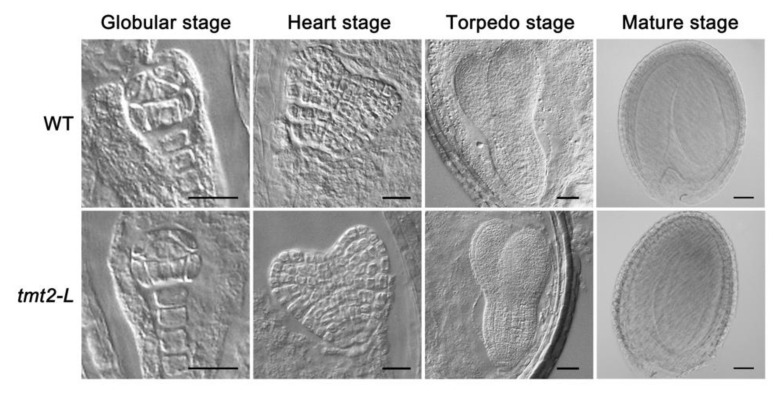
Embryogenesis observation of the WT and *tmt2-L* mutant by DIC microscope. Scale bars = 10 μm. DIC: differential interference contrast.

Seeds harvested from *tmt2-L* constantly produced a large number of UnG and *tmt2-S* offspring, generation after generation, prompting us to postulate that there might be some defects in seed development. Seed development is composed of two processes: embryogenesis and seed maturation. We first examined the embryogenesis process of the WT and *tmt2-L* after pollination at several time points. Embryos at different stages of the WT and *tmt2-L* were sampled and observed with a differential interference contrast (DIC) microscope. The early globular, heart, torpedo, and maturation stages of WT and *tmt2-L* embryos were analyzed, and we found that there were no obvious differences in embryogenesis between WT and *tmt2-L* plants (Figure 5).

Storage materials accumulated in seeds are mainly presented as lipids, sugars, and proteins [33]. Lipids are major storage materials in Arabidopsis seeds, and usually, oil constitutes up to 40% of the seed’s fresh weight. Lipid catabolism deficiencies can lead to arrested development during germination and early seedling growth [7,34,35]. By analyzing lipid droplets in embryos at the torpedo stage and lipid contents in seeds, we found that lipid droplets and lipid contents of *tmt2-L* were almost identical to those of the WT (Supplemental Figure S8). No defect in lipid accumulation was found in the *tmt2* mutant.

After the embryogenesis study, we performed a detailed analysis of seed maturation (Supplemental Figure S9). At first, we examined all siliques at the development stage from the WT and *tmt2-L* after about 40 days of growth, and we found that siliques at the upper, middle, and lower positions in the WT and *tmt2-L* main stems behaved almost identically. No difference was observed in silique appearance and length between the WT and *tmt2-L* (Supplemental Figure S9A). After the siliques matured and dried out, we found that the number of seeds per silique, weight per 1000 seeds, volume per 1000 seeds, and seed weight per plant were almost the same between the WT and *tmt2-L* (Supplemental Figure S9B–E). The lack of TMT2 did not affect seed maturation in *tmt2-L*, which is also consistent with the analysis that there was no defect in the embryogenesis process (Figure 5).

A previous study showed that *TMT1* overexpression plants had improved seed weight, lipid, and protein contents [17]. We hypothesized whether the overexpression of TMT2 would have a similar effect on Arabidopsis development. In order to explore the possibility, we transformed *P_35S_::TMT2* into WT plants to generate the overexpressing line *OE-TMT2*. The results showed that the overexpressing line *OE-TMT2*, which carried a much-elevated level of *TMT2* expression, grew the same as the WT and had a similar weight per 1000 seeds, a similar seed volume, and similar lipid and protein contents with the WT (Supplemental Figure S10).

In contrast to its vital roles in germination and initial development, it seemed that TMT2 was not essential for vegetative growth and reproductive processes in Arabidopsis once *tmt2* developed into *tmt2-L* plants. Neither the deficiency of TMT2 nor the overexpression of *TMT2* significantly affected the growth and development after the initial stage in Arabidopsis.

### 2.6. The Development of True Leaves Was Arrested in the tmt2 Mutant

The *tmt2-L* plants grew normally in the vegetative growth, embryogenesis, and seed maturation process. All data showed that the growth defects of *tmt2* mainly occurred at the stage of germination and early seedling development, which are highly energy-consuming and require plenty of substances converted from cotyledons [36].

**Figure 6:**
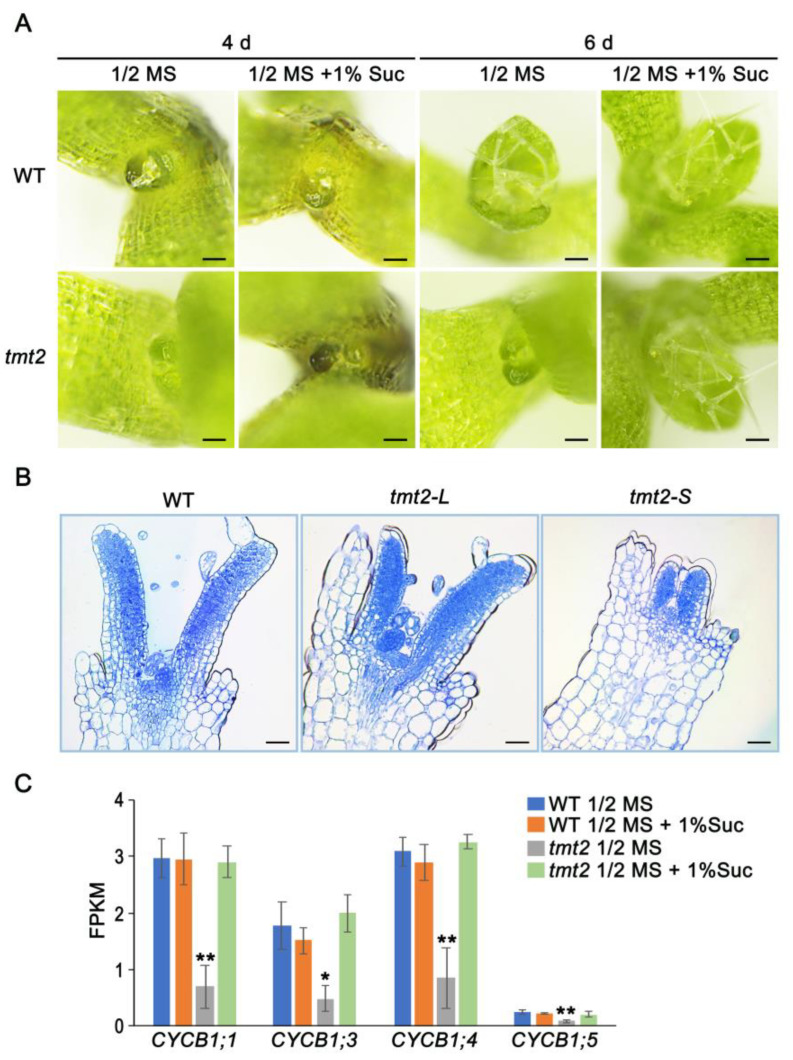
True leaf development and SAM formation of WT and *tmt2* seedlings. (**A**) Observation of true leaves of WT and *tmt2* seedlings on 1/2 MS media and 1/2 MS media with 1% sucrose on the fourth day and sixth day post-germination. Scale bars = 200 μm. (**B**) Longitudinal sections of SAM of WT, *tmt2-L*, and *tmt2-S* seedlings. Scale bars = 50 μm. (**C**) The expression levels of mitotic quiescent reporter genes *CYCB1;1*, *CYCB1;3*, *CYCB1;4*, and *CYCB1;5* in WT and *tmt2* seedlings grown on 1/2 MS media without sucrose and 1/2 MS media with 1% sucrose, separately. Error bars represent the SD of three biological replicates. Significant differences are indicated as *, *p* < 0.05, **, and *p* < 0.01 by the Student’s *t*-test. SAM: shoot apical meristem, *CYCB1;1*: cyclin B1;1, *CYCB1;3*: cyclin B1;3, *CYCB1;4*: cyclin B1;4, *CYCB1;5*: cyclin B1;5.

To gain an insight into abnormal phenotypes of *tmt2*, we closely analyzed the initial developing stage of the WT and *tmt2*. On the fourth day post-germination, no true leaves could be clearly observed from the WT and *tmt2* grown on 1/2 MS media or 1/2 MS media with 1% sucrose. On the sixth day post-germination, the WT grew remarkably visible true leaves on 1/2 MS media with or without 1% sucrose, while *tmt2-S* grew true leaves only on 1/2 MS media with 1% sucrose but not on 1/2MS media without sucrose (Figure 6A). We compared shoot apical meristems (SAM) among the WT, *tmt2-L*, and *tmt2-S* seedlings. As expected, the SAMs of *tmt2-L* seedlings looked like those of the WT, and both WT and *tmt2-L* SAMs could further develop into true leaves. However, SAMs of *tmt2-S* were not able to develop further into true leaves (Figure 6B). The deficiency of true leaf development of *tmt2-S* is likely due to the defect in cell division, which is an essential step for leaf organogenesis. We examined mitotic quiescent reporter genes cyclin B1;1 (*CYCB1;1)*, *CYCB1;3*, *CYCB1;4*, and *CYCB1;5* in the initial developing stage. RNA-seq results revealed that the expressions of mitotic reporter genes in SAMs were highly suppressed in *tmt2* on 1/2 MS media; on the contrary, they were restored into WT-like levels when *tmt2* was grown on 1/2 MS media with 1% sucrose (Figure 6C).

### 2.7. Functional Cotyledons and Sugar Could Sustain the Early Seedling Growth of tmt2-S

**Figure 7:**
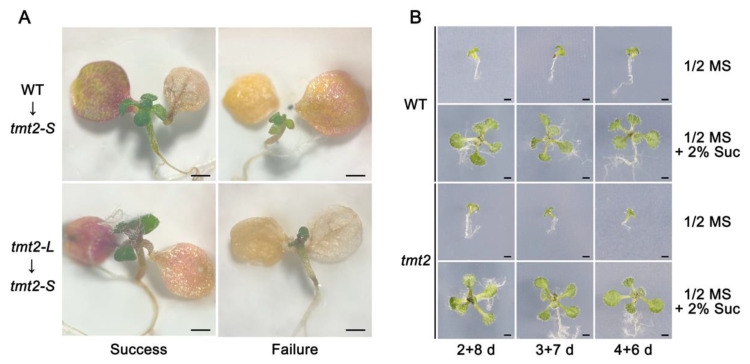
Sugar from cotyledons promoted seedling growth. (**A**) The cotyledons of WT and *tmt2-L* seedlings were transplanted to *tmt2-S* seedlings without cotyledons. WT→*tmt2-S* indicates that the cotyledons of WT seedlings were transplanted to the petioles of *tmt2-S*. *tmt2-L*→*tmt2-S* implies that the cotyledons of *tmt2-L* seedlings were transplanted to the petioles of *tmt2-S*. Successful attachment of the cotyledons to the petioles was recorded as successful micrografting, while the event where the cotyledons failed to attach to the petioles was recorded as failed micrografting. Successful and failed micrografting results were shown in two columns. Micrografting plants were photographed after 12 days of growth. Scale bars = 1 mm. (**B**) Sucrose supply supported seedling growth of the WT and *tmt2* without the cotyledons. Here, 2 + 8 d means that the cotyledons of seedlings were cut on the 2nd growth day and then grew for another 8 days. Additionally, 3 + 7 d and 4 + 6 d have the same meanings as 2 + 8 d. Three repeats were performed. Scale bars = 1 mm.

Dysfunction in cotyledons or deficiency in SAM would cause seedlings to fail to produce true leaves [34,37]. From Figure 1, we know that the cotyledons of *tmt2-S* were more dark-green in color and smaller than those of the WT and *tmt2-L*, suggesting dysfunction in the *tmt2-S* cotyledons. Therefore, we conducted a cotyledon micrografting experiment to test the influence of cotyledons on true leaf growth. Cotyledons of 5-day-old *tmt2-S* seedlings on 1/2 MS media were removed from the plantlets, which served as the grafting recipient. Cotyledons of the donors, the WT and *tmt2-L,* were transplanted onto the petioles of *tmt2-S* seedlings without cotyledons. After 12 days of micrografting, *tmt2-S* was able to grow true leaves after successful micrografting with the cotyledons from WT and *tmt2-L* seedlings. When micrografting failed, *tmt2-S* still grew abnormally as before (Figure 7A). This suggested that the abnormal growth of *tmt2-S* seedlings was due to the defects in the cotyledons, which could lead to an insufficient mobilization of the sugars to the seedlings during early seedling development.

The cotyledons of the WT or *tmt2-L* could enable *tmt2-S* to grow true leaves. It is interesting to identify the components from cotyledons that support true leaf growth, which are likely to be sugars. In order to test this postulation, we removed cotyledons from WT and *tmt2-S* seedlings grown for 2 days, 3 days, or 4 days on 1/2 MS media, and transferred these plantlets without cotyledons onto 1/2 MS media without sucrose and 1/2 MS media with 2% sucrose to continue their growth. The WT and *tmt2-S* seedlings without cotyledons all failed to grow on 1/2 MS media without sucrose. After continuously being grown on 1/2 MS with 2% sucrose, both WT and *tmt2-S* seedlings without cotyledons showed normal growth with true leaf development (Figure 7B and Supplemental Figure S11). These results implied that the effect of sugar could restore *tmt2-S* to normal growth, and the cotyledons deliver sugar to the seedlings, which is essential for plant growth. Noticeably, since we excluded un-germinated seeds and very small *tmt2-S* plants in this experiment, it seems that the rescuing effect of 2% sucrose on cotyledon-defoliated *tmt2-S* plants (Supplemental Figure S11) was better than that on *tmt2* seeds (Figure 3).

### 2.8. Global Gene Expressions, Especially Energy, Hormones, and TOR Pathways, Were Altered in tmt2 Due to Sugar Shortage

The defects of *tmt2* mainly occurred at the stage of germination and initial development. In order to better understand the critical roles of TMT2, it is necessary to dissect the gene expression activities of the *tmt2* mutant at the early developmental stage. Therefore, we carried out a detailed RNA-seq analysis on four different samples, including the WT on 1/2 MS media, the WT on 1/2 MS media with 1% sucrose (WT-Suc), *tmt2* on 1/2 MS media (*tmt2-S*), and *tmt2* on 1/2 MS media with 1% sucrose (*tmt2-L-Suc*). As for the WT, WT-Suc, and *tmt2-L-Suc* samples, we collected seedlings on the fourth day post-germination when the plants started to grow true leaves, because we believed that the gene expression at this point would reflect the gene transcriptional requirement for true leaf development. Since *tmt2-S* grows slower than other samples, we collected *tmt2-S* seedlings on the sixth day post-germination to reflect a similar growth stage.

**Figure 8:**
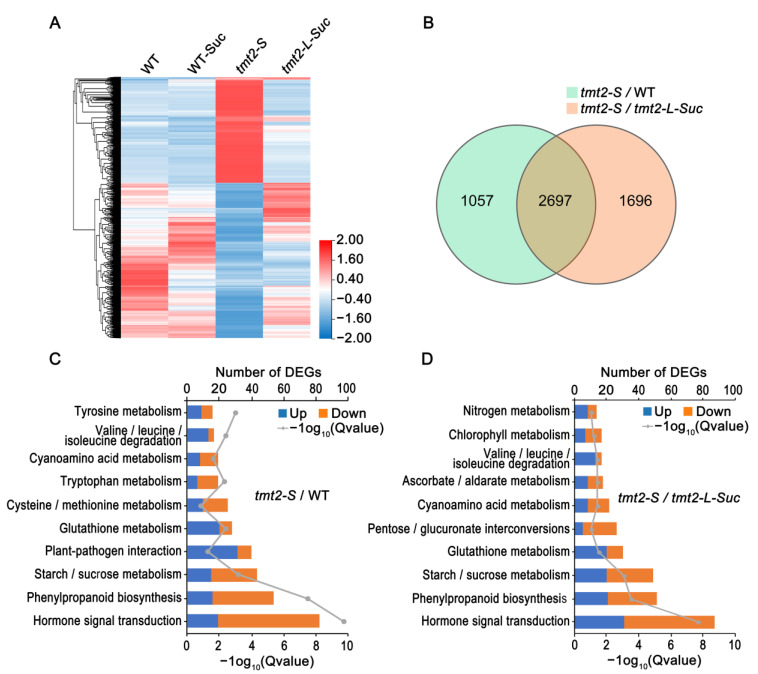
Transcriptome analysis of the WT, WT-Suc, *tmt2-S*, and *tmt2-L-Suc* plants. (**A**) Heatmap of DEGs expressed in WT, WT-Suc, *tmt2-S*, and *tmt2-L-Suc*. WT indicates 4-day-old WT seedlings on 1/2 media, WT-Suc indicates 4-day-old WT seedlings on 1/2 media with 1% sucrose, *tmt2-S* indicates 6-day-old *tmt2* seedlings on 1/2 media, and *tmt2-L-Suc* indicates 4-day-old *tmt2* seedlings on 1/2 media with 1% sucrose. (**B**) Venn diagram of *tmt2-S*/WT (green) and *tmt2-S*/*tmt2-L-Suc* (pink) DEGs. (**C**) The KEGG pathway enrichment histogram of *tmt2-S*/WT DEGs. (**D**) The KEGG pathway enrichment histogram of *tmt2-S*/*tmt2-L-Suc* DEGs. DEGs: differentially expressed genes.

Not surprisingly, the heatmap analysis of the RNA-seq data showed that the global gene transcription pattern in *tmt2-S* was remarkably distinguished from those in the WT, WT-Suc, and *tmt2-L-Suc*, reflecting the severe phenotypical defect of *tmt2-S* (Figure 8A). The WT, WT-Suc, and *tmt2-L-Suc* samples showed similar gene expression patterns with some remarkable differences. The transcriptional differences between the WT and WT-Suc indicated that the addition of sucrose caused a substantial gene expression change in response to the environmental sucrose. The transcript comparison between the WT, *tmt2-S*, and *tmt2-L-Suc* samples showed that the addition of sucrose reprogrammed the gene expression in *tmt2*, consequently restoring *tmt2* seedlings to a normal seedling development (Figure 3 and Figure 8A). Based on the criteria of |log_2_FC| ≥ 1.2 and Q value ≤ 0.05, differentially expressed gene (DEG) enrichment analysis showed that there are 3754 DEGs in the comparison of *tmt2-S*/WT and 4393 DEGs in the comparison of *tmt2-S*/*tmt2-L-Suc,* respectively (Figure 8B). According to the KEGG pathway analysis, we found that the DEG enrichment of *tmt2-S*/*tmt2-L-Suc* is similar to that of *tmt2-S*/WT, mainly reflecting the changes in the gene expression levels of plant hormone signal transduction, sugar, and amino acid biosynthesis and catabolism pathways (Figure 8C,D). The volcano map indicated the most significantly differentially expressed genes between WT and *tmt2-S* (Supplemental Figure S12A), among which *FLOT2*, *IAN8*, and *LHT7* were the top three up-regulated genes, and *SAUR22*/*23*/*26* were the top three down-regulated genes, *HR4*, *RAP2.6L*, and *DTX1* were the top three credible up-regulated genes, and *GASA6*, *JAL22*, *and PIN7* were the top three credible down-regulated genes (Supplemental Figure S12A). The expression levels of the top three up-regulated genes *SAUR22*/*23*/*26* were also confirmed by a qRT-PCR assay, showing that the qRT-PCR results of the transcript were similar to those shown in the RNA-seq analysis (Supplemental Figure S12B). The DEGs of *tmt2-S*/WT were classified into three GO sets, including biological process (BP, 1867 genes), molecular function (MF, 1161 genes), and cellular component (CC, 312 genes). The top six significantly altered groups of each GO category are summarized in Supplemental Figure S12C, suggesting that the deficiency of *TMT2* resulted in significant alterations in gene expression patterns of these processes.

**Figure 9:**
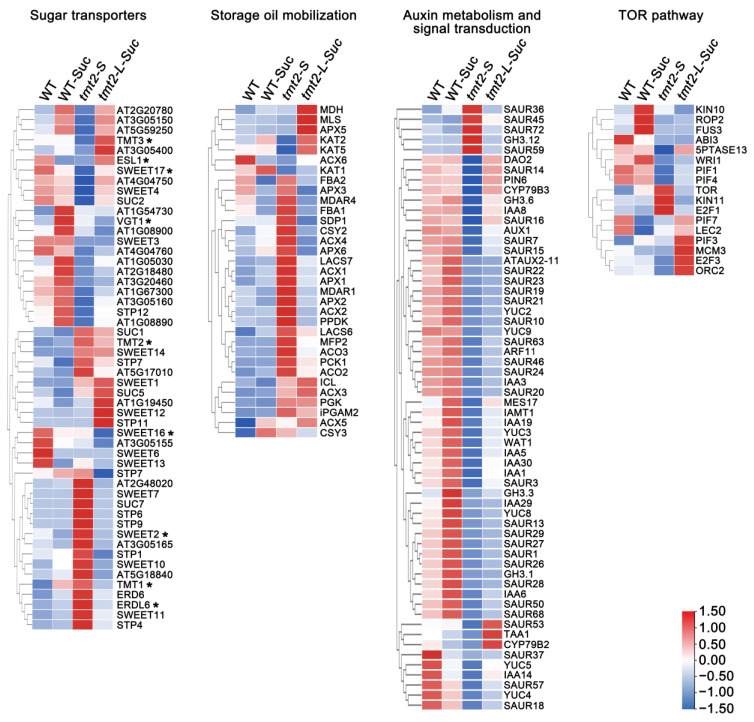
Heatmap of gene expressions for sugar transporter, storage oil mobilization, auxin metabolism and signal transduction, and TOR pathway. Stars indicate the sugar transporters in the tonoplast. TOR: target of rapamycin.

Based on the global gene expression analysis in the WT, WT-Suc, *tmt2-S*, and *tmt2-L-Suc* samples, we concluded that the following genes were tightly related to developmental defects in the *tmt2* mutant, including genes encoding sugar transport proteins, genes involved in storage oil mobilization, genes for the auxin metabolism and signal transduction, and genes for the TOR pathway (Figure 9). Among 12 vacuolar sugar transporters, *TMT1*, *SWEET2*, and *ERDL6* were up-regulated in *tmt2-S* and suppressed in *tmt2-L-Suc*, suggesting that they could play a functional role similar to TMT2 (Figure 9, Supplemental Figure S13). Storage oil mobilization is critical for germination and initial seedling development in Arabidopsis [7,35]. We found that the genes for key enzymes in storage oil mobilization were induced to a very high level in *tmt2-S* but then returned to a WT expression level in *tmt2-L*, demonstrating that plant cells tried to convert lipid into sucrose to meet the high carbohydrate requirement for seedling development. As for auxin metabolism and signaling pathways, the expressions of most genes were suppressed in *tmt2*. Interestingly, only a small portion of auxin-related genes, such as dioxygenase for auxin oxidation 2 (*DAO2*), small auxin up RNA 14 (*SAUR14*), PIN-formed 6 (*PIN6*), cytochrome P450, family 79, subfamily B, polypeptides 3 (*CYP79B3*), Gretchen Hagen 3.6 (*GH3.6*), etc., could be restored to the WT level by sucrose application (Figure 9 and Supplemental Figure S13). Notably, *SAUR36*, *SAUR45*, *SAUR59*, *SAUR72*, and *GH3.12* were significantly up-regulated in *tmt2-S* and could be restored to the WT transcription level through the addition of sucrose. Another notable event is that *SAUR53*, tryptophan aminotransferase of Arabidopsis 1 (*TAA1*), and *CYP79B2* were remarkably up-regulated in *tmt2-L-Suc* compared to the WT, WT-Suc, and *tmt2-S* (Figure 9 and Supplemental Figure S12). Meanwhile, TOR pathway genes showed obvious expression differences in the WT and *tmt2*. Key components in TOR pathways, including *TOR*, SNF1 kinase homolog 11 (*KIN11*), and E2F transcription factor 1 (*E2F1*), were up-regulated in *tmt2-S* but were restored to WT expression levels in *tmt2-L-Suc* (Figure 9 and Supplemental Figure S13). However, other TOR pathway genes did not show this pattern, indicating that genes in the TOR pathways played complex roles in the *tmt2* mutant.

### 2.9. The Abnormal Phenotype of the tmt2 Mutant Was Not Rescued by IAA

**Figure 10:**
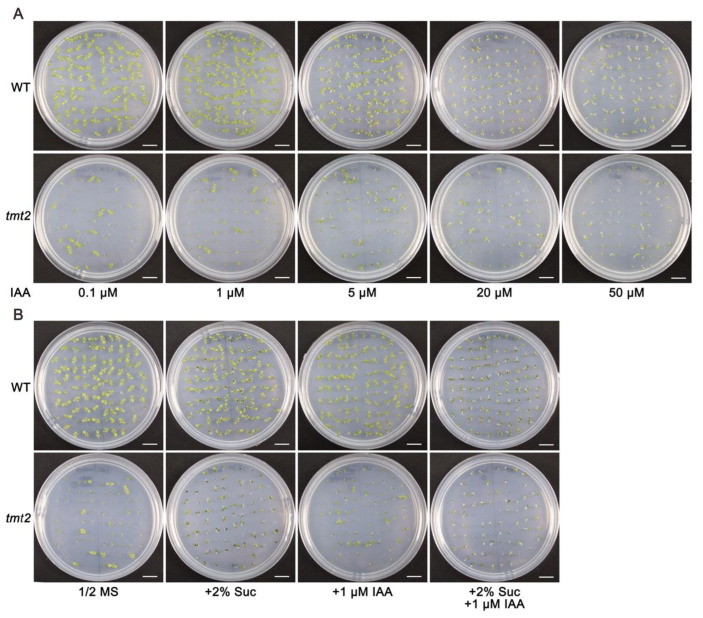
Initial growth of WT and *tmt2* seedlings on 1/2 MS media with IAA alone and with IAA and sucrose. (**A**) Initial growth of 7-day-old WT and *tmt2* seedlings on 1/2 MS media with different contents of IAA. (**B**) Initial growth of 7-day-old WT and *tmt2* seedlings on 1/2 MS media, 1/2 MS media with 2% sucrose, 1/2 MS media with 1 μM IAA, and 1/2 MS media with 2% sucrose and 1 μM IAA. Each experiment is a representative of three repeats. Scale bars = 1 cm.

Since most auxin metabolism and signaling pathway-related genes were down-regulated, we were interested in exploring whether the addition of IAA could rescue the abnormal phenotypes of *tmt2* (Figure 9). In order to test this speculation, the WT and *tmt2* were grown on 1/2 MS media supplied with different contents of IAA. The results showed that the *tmt2* mutant did not change its growth with the addition of IAA (Figure 10A). In order to investigate whether IAA had a positive effect on *tmt2* development in the presence of the application of sugar, we supplied both IAA and sucrose into 1/2 MS media and found that only sugar could rescue *tmt2*, and the exogenous IAA is not beneficial to the rescuing effect of sucrose on the *tmt2* mutant (Figure 10B). In conclusion, the exogenous IAA was unable to compensate for the phenotypes of *tmt2*, which indicated that the auxin pathway is essential but not sufficient for the sugar-triggered initial plant development.

## 3. Discussion

Sugars are energy substances and signal molecules that play indispensable functions in plants and other organisms. Sugar transport from the source to the sink is accomplished by a variety of sugar transporters. Therefore, understanding the function and regulation of sugar transporters is of great importance for improving plant biomass production or seed yield in future agriculture [38,39]. Here, we found that a vacuolar sugar transporter, TMT2, plays a vital role in germination and initial seedling development, as shown by the fact that *tmt2* plants produced a large amount of un-germinated seeds (UnG) and seedlings without true leaves *(tmt2-S*) (Figure 1C,D). Severe defects of *tmt2* in germination and early seedling development demonstrated that TMT2 plays a vital role in cotyledon development and mobilizing sugars from the cotyledons to seedlings.

The abnormal phenotypes of *tmt2* were quite obvious, which made us wonder why they had not been reported in several previous studies. Wormit et al. (2006) reported that the *tmt2* mutant did not exhibit any distinctive phenotypes when grown on 1/2 MS media with 1% sucrose or in soil under standard growth conditions [19]. Wingenter et al. (2010) observed that the *tmt1*/*tmt2* double mutant grew slower than the WT within the first 15 days in soil but was able to catch up with the WT afterward [17]. The main discrepancy between this work and previous reports could be explained by the fact that our growth condition is 1/2 MS media without 1% sucrose, and sucrose was able to largely rescue the growth defects of *tmt2* (Figure 1 and Figure 3). Once *tmt2* developed into normal-looking *tmt2-L* plants, it was very difficult to distinguish it from the WT (Figure 1H). Interestingly, the germination rate and initial growth of *tmt2* grown in the soil were similar to *tmt2* grown on 1/2 MS media with 1% sucrose and not similar to 1/2 MS media only (Figure 1A,C, and Supplemental Figure S2). Our results suggested that it would be a better choice to grow plants without a sugar supply when mutants lacking sugar transporters were studied.

A typical penetrance was observed in the *tmt2* mutant, that offspring constantly produced UnG seeds, *tmt2-S* plants, and *tmt2-L* plants. As a vacuolar transporter, TMT2 transports sugar across the tonoplast [17,18]. In the absence of TMT2, plants have to use alternative means to sustain cotyledon development and mobilize sugars from the cotyledons to seedlings. There are 12 sugar transporters residing on the Arabidopsis tonoplast, including 3 VGTs, 3 TMTs, 3 SWEETs, SUC4, ERDL6, and ESL1 [16]. Notably, *TMT1*, *SWEET2*, and *ERDL6* showed similar expression patterns as *TMT2*, suggesting that they may partially complement the deficiency of *TMT2* (Figure 9). The sugar content of *tmt2-L* cotyledons was much higher than that of the WT, while *tmt2-S* cotyledons maintained a similar sugar level as the WT. This suggested that when the cotyledon sugar content reaches a certain threshold concentration to meet the developmental requirements, the *tmt2* mutant can grow as well as the WT (Figure 2). Plant germination and initial development highly rely on substances and signals from the cotyledons [40,41], and the sugar supply can largely sustain the initial development without the presence of cotyledons (Figure 7). Only a high concentration of sugar in the cotyledons would enable seedlings to undergo normal development in *tmt2*. If *tmt2* plants kept a WT-similar sugar content, then true leaf development would be arrested because of its incapability to transport sugar from the cotyledons to seedlings due to the TMT2 malfunction.

In Arabidopsis, oil is the major storage compound in seeds. However, the oil stored in the cotyledons does not directly move into the seedlings to support growth. The oil needs to be transformed into sugar by gluconeogenesis to be further utilized by the seedlings [7]. Mutants defective in storage oil mobilization usually show impaired germination and arrested true leaf development, and providing sucrose can rescue their impaired germination and post-germinative growth [42]. Lipid droplet and lipid content analysis showed that there is the same lipid accumulation in the WT and *tmt2* (Supplemental Figure S9), and RNA-seq showed that the genes for storage oil mobilization were up-regulated in *tmt2* plants (Figure 9). It is very likely that the TMT2 deficiency demands and accelerates the transformation of sugars from oil, resulting in high sugar content and normal growth in *tmt2-L* plants.

In contrast to severe defects in germination and initial seedling growth, the *tmt2* mutant showed WT-like normal vegetative and reproductive growth once it developed into *tmt2-L* plants. Photosynthetic activity, vegetative growth, flower formation, seed maturation, and lipid content of *tmt2-L* plants are indistinguishable from those of the WT (Supplemental Figures S7–S9 and Figure 5). Neither the deficiency of TMT2 nor the overexpression of TMT2 significantly affected the growth and development after the initial stage in Arabidopsis. Previous studies also reported that *tmt2* plants did not show visible phenotypical differences compared to the WT [17,18,19], which was in agreement with our observation. There are two possibilities for the dispensability: either TMT2 is not important for normal growth after the initial growth, or its function could be complemented by the redundancy of other vacuolar sugar transporters.

However, we did find that *tmt2-L* plants were more sensitive to high sugar concentrations compared to the WT (Supplemental Figure S5). Sugar is an essential energy substance and signaling molecule to sustain plant life. Excessive cellular sugar is also a severe stressor for plants, and maintaining sugar homeostasis is vital for normal development. Vacuolar sugar transporters are the major players in maintaining the cellular sugar homeostasis of plants by storing excess sugar in the vacuoles and unloading it into the cytosol when necessary. The hypersensitivity of *tmt2-L* plants to high concentrations of sugar illustrated that TMT2 is important for plant cells to maintain their sugar homeostasis. This is also in agreement with previous studies showing that *tmt2* plants usually showed growth defects under stress conditions [17,18,19].

There are several vacuolar sugar transporters in Arabidopsis, and they could function in different ways, such as by transporting different sugars in different orientations, inward and outward of the tonoplast. It has been reported that TMT2 could be a proton-coupled antiporter in charge of loading glucose and sucrose into the vacuoles in leaf tissues [17,18]. The higher sensitivity of *tmt2-L* to high concentrations of sugar supports this idea (Supplemental Figure S5). The absence of TMT2 in plants would hurdle storing sugar in the vacuoles; therefore, the sugar level would be kept at a high level, which would lead plant cells to high sugar stress conditions. Our results supported the idea that TMT2 can be a sugar importer to load sugar into the vacuoles.

It is well known that plant hormones play crucial roles in plant growth and development. Auxin triggers cell proliferation and differentiation in SAM, which is essential for true leaf development [43]. In the past year, the crosstalk between sugar and auxin has been gradually understood in plant development [44,45]. From the results of the RNA-seq analysis, we knew that IAA pathway genes were significantly altered in the *tmt2-S* mutant. In contrast, the IAA application is not enough to rescue the abnormal phenotypes of the *tmt2* mutant (Figure 9 and Figure 10). This indicated that auxin signaling pathways are essential but not sufficient for the sugar-triggered initial plant development. This is in agreement with the hypothesis that the addition of auxin cannot rescue developmental defects due to a glucose deficiency [37]. We noticed that the down-regulated IAA genes caused by the TMT2 deficiency could not be reversed through the addition of sucrose (Figure 9), indicating that there is a big difference between sugar signaling and auxin signaling, and the crosstalk between sugars and auxin is very complicated. This supports the hypothesis that glucose and auxin can synergistically or antagonistically regulate global gene expression in cells.

The TOR signaling pathway is crucial for promoting cell proliferation and plays an important role in modulating the availability of nutrients and energy for optimal growth and development [37,44]. It has been shown that the TOR kinase is a major glucose signaling mediator to control plant growth and development [46,47]. In the *tmt2* mutant, TOR pathway genes showed clearly altered expression levels. For example, key components in TOR pathways, including *TOR*, *KIN11*, and *E2F1*, were up-regulated in *tmt2-S* but were restored to WT expression levels in *tmt2-L*. However, not all TOR genes showed this pattern, indicating that genes in the TOR pathways played complex roles in the *tmt2* mutant.

## 4. Materials and Methods

### 4.1. Primers

All the primers used in this study are listed in Supplementary Table S1.

### 4.2. Plant Materials and Growth Conditions

The T-DNA insertional *tmt2* mutant line, SAIL_124_H03, with the Columbia-0 genetic background, was obtained from the Nottingham Arabidopsis Stock Center (https://arabidopsis.info/BasicForm) on 20 December 2019. The mutant was verified as a homozygous mutant by PCR, and its T-DNA insertion site was determined by DNA sequencing.

After surface sterilization with 70% ethanol for 5 min and 50% sodium hypochlorite with 0.05% tween for 10 min, Arabidopsis seeds were washed 4 times and sown on 0.8% (*w*/*v*) agar media (pH 5.7) containing 1/2 MS with or without sugar. Seeds sown in nutrient soil (Pindstrup, Midtjylland, Denmark) or plated on 1/2 MS media were transferred into the growth chamber or greenhouse after 2 days of vernalization and were cultivated under normal conditions (23 ℃, 16 h light/8 h dark photoperiod, and 100 μmol·m^−2^·s^−1^ light intensity). To analyze the rescuing effect of sucrose, 5-day-old *tmt2-S* seedlings grown on 1/2 MS media were transferred onto a new 1/2 MS media and 1/2 MS media with 1% sucrose for another 7-day growth. To test the sensitivity to a higher sugar content, 5-day-old WT and *tmt2-L* seedlings grown on 1/2 MS media with 1% sucrose were transferred onto 1/2 MS media with 3% sucrose for another 7 days of growth.

Seeds without radicals were counted as un-germinated seeds (UnG), seedlings without obvious true leaves after 4 days were considered abnormal seedlings (*tmt2-S*), and normally developing seedlings similar to the WT were recorded as *tmt2-L*.

### 4.3. RNA Extraction, RT-PCR, and Quantitative RT-PCR Assays

Total RNA of Arabidopsis different tissues was extracted using the RNAprep Pure Plant kit (Tiangen, Beijing, China). A total of 1 μg of total RNA was reversely transcribed into cDNA using the Transcript One-step gDNA Removal and cDNA Synthesis Supermix kit (TransGen, Beijing, China) according to the manufacturer’s instructions. RT-PCR was used to examine the expression levels of target genes, with *ACTIN2* serving as the reference gene. Gene expression analysis was also performed by qRT-PCR with FastStart Universal SYBR Green Master (Yeasen, Shanghai, China), with *ACTIN8* serving as the reference gene.

### 4.4. Vector Construction and Plant Transformation

The full-length coding sequence of *TMT2* was amplified by PCR with cDNA as the template. The HA tag was ligated to the 3′ end of the amplified product to examine the TMT2 protein level using the anti-HA antibody. The fused *TMT2-HA* gene was cloned into the pRI101-AN vector driven by the *CaMV-35S* promoter. The 1649 bp DNA fragment before the start code of *TMT2* was amplified by PCR from genomic DNA and was ligated in front of the fused gene *TMT2-HA*. The obtained *P_TMT2_::TMT2-HA* was cloned into the pRI101-AN vector with the *CaMV-35S* promoter removed. For the complementation assay, *Agrobacterium tumefaciens* carrying the recombinant vectors were transformed into *tmt2* plants by the floral dip method [48,49]. For the overexpression experiment, the recombinant vector pRI101-AN*-P_35S_::TMT2-HA* was transformed into WT plants. T3 generation homozygous lines derived from three independent T1 transgenic lines were applied for further analysis.

### 4.5. Cloning of P_TMT2_::GUS and Histochemical GUS Staining

The 1649 bp DNA fragment before the start code of *TMT2* was inserted into the pCAMBIA1305 vector containing the *GUS* gene and named pCAMBIA1305*-P_TMT2_::GUS*. The construct was transformed into the WT plants through *Agrobacterium tumefaciens* to generate transgenic plants expressing the P_TMT2_::GUS fused protein. A total of 37 independent transgenic lines were analyzed for GUS activities in T3 generation. Histochemical GUS staining assay was carried out according to Jefferson et al. (1987) and Chang et al. (2013) with minor modifications [50,51]. Tissues of transgenic plants at different stages grown on 1/2 MS media or in soil were immersed in 90% acetone and incubated on ice for 20 min. The samples were rinsed three times with the washing solution (50 mM sodium phosphate buffer (pH 7.0), 0.5 mM potassium ferrocyanide, and 0.5 mM potassium ferricyanide). Then, the staining solution (100 mM sodium phosphate buffer (pH 7.0), 0.5 mM potassium ferrocyanide, 0.5 mM potassium ferricyanide, 1% Triton X-100, 10 mM EDTA, and 1 mM 5-bromo-4-chloro-3-indolyl-β-D-glucuronic acid (X-Gluc)) was added to the samples. After vacuuming for 2 min, the samples were incubated overnight at 37 °C and washed with 15% and 30% ethanol in turn, and the resulting GUS staining pattern was recorded with a dissecting microscope (Nikon, Tokyo, Japan).

### 4.6. Sugar Quantification

The sugar quantification was performed using high-performance anion-exchange chromatography (HPAEC) on a CarboPac PA-1 column using a pulsed amperometric detector (Thermo Scientific, Waltham, MA, USA) according to Poschet et al. (2011) [29]. Aerial parts of WT, *tmt2-S*, and *tmt2-L* seedlings grown on 1/2 MS media were frozen in a −80 °C freezer until use. Approximately 50 mg samples were immersed in 80% alcohol in a tube and shocked at 50 °C for 2 h. The sample was diluted by adding ddH_2_O and centrifuged at 10,000 rpm for 3 min, and then the supernatant was used for quantification.

### 4.7. Subcellular Localization of TMT2 Protein

The full-length coding sequence of *TMT2* was inserted into the pCAMBIA2300 vector with the *eGFP* gene and named pCAMBIA2300*-P_35S_::TMT2-eGFP.* One-month-old tobacco leaves (*Nicotiana tabacum*) were backside-injected with *Agrobacterium tumefaciens* carrying the recombinant vector. After one day of shade growth and one day of light growth, protoplasts were extracted from the injected leaves, as described by Yoo et al. (2007) [52]. The intact protoplasts were dropped on the slide and observed under a confocal microscope (Leica, Wetzlar, Germany). GFP was excited at 488 nm and detected using a 495–545 nm emission filter, while chlorophyll was excited at 552 nm and detected using a 580–630 nm emission filter.

### 4.8. Chlorophyll Fluorescence Measurements

Chlorophyll fluorescence was measured with a Dual-PAM-100 chlorophyll fluorometer (Heinz-Walz, Nuremberg, Germany) using 3-week-old plant leaves. After dark treatment for 30 min, the intact leaves were illuminated with 300 ms saturating light (20,000 μmol·m^−2^·s^−1^). The intensity of actinic light was set to 214 μmol·m^−2^·s^−1^. The gradient photosynthetically active radiation (PAR) was set, and rapid light curves were measured. The maximum photochemical efficiency of PSII (Fv′/Fm′) in any light intensity was calculated as (Fm′ − Fo′)/Fm′. The effective PSII quantum yield Y(PSII) was defined as (Fm′ − Fs)/Fm′. The coefficient of photochemical quenching (1− qP) was equal to 1 − (Fm′ − Fs)/(Fm′ − Fo′), and the coefficient of nonphotochemical quenching (NPQ) was defined by the formula (Fm − Fm′)/Fm′ [53].

### 4.9. Characterization of Embryogenesis

For the characterization of embryogenesis of WT and *tmt2-L* plants, ovules from different stages were selected as testing samples. Selected ovules were cleared with Hoyer’s solution and dark-treated at room temperature for 2–12 h according to the maturation degree of the ovules [54]. Different embryo developmental stages were examined using a DIC microscope (Leica, Wetzlar, Germany).

### 4.10. Lipid Droplet Observation

Seed embryos in the torpedo stage were obtained from siliques with a tweezer under a dissecting microscope (Leica, Wetzlar, Germany), stained in tubes with Nile red solution (10 μg/mL in 0.1 M Tris-HCl buffer (pH 8.0)) for 30 min, and then washed with 0.1 M Tris-HCl buffer (pH 8.0) for 10 min. Fluorescence of Nile red dye was observed with a wavelength of 488 nm for excitation and 550 to 650 nm for emission.

### 4.11. Cotyledon Micrografting

Seeds of the WT and *tmt2* mutant were planted on 1/2 MS media and stratified for 2 days. The cotyledon micrografting experiment was conducted according to the method of Bartusch et al. (2020) with minor modifications [55]. Cotyledons of *tmt2-S* seedlings grown on 1/2 MS media for 5 days were removed, and cotyledon-defoliated seedlings served as the grafting recipient. Then, cotyledon-defoliated seedlings were transferred to the nylon membrane in a dish containing two round moistened filter papers with forceps. Cotyledons of the WT and *tmt2-L* grown on 1/2 MS media for 5 days were cut off as the grafting donor and transferred to the recipient seedlings. Donor cotyledons were transplanted to the petiole of recipient seedlings gently under a dissecting microscope. After the micrografting, the dishes were sealed with parafilm, and grafted plants continued to grow in the growth chamber. On the 7th day of micrografting, the grafted plants were evaluated under a dissecting microscope and photographed. Well-attached cotyledons were considered successful micrografting, and detached cotyledons were counted as failed micrografting.

### 4.12. RNA-Seq Analysis

Total RNA was extracted from above-ground plant tissues of WT and *tmt2* on 1/2 MS media without sucrose and 1/2 MS media with 1% sucrose. Each sample has three repeats. The qualified total RNA was used for library construction. DNA in total RNA was digested by DNase I, and then mRNA with PolyA tail was enriched by oligo (dT) magnetic beads. The mRNA was subsequently fragmented by the fragmentation buffer. After double-stranded cDNA was synthesized by reverse transcription with random primers, the ends of double-stranded cDNA were flattened and connected to the adaptors. PCR amplification was performed by specific primers, and PCR products were denatured to single strands, which were subsequently cyclized by bridge primers. RNA-deep sequencing was performed on the MGISeq 2000 platform with a read length of 150. Raw data with low-quality reads, contaminated adaptors, and high content of unknown base N were filtered to obtain clean data, and clean reads were aligned to the *Arabidopsis thaliana* reference genome TAIR10.1 through Bowtie2. The gene expression levels were calculated by RSEM, and the differential expressed genes were collected based on |log_2_FC| ≥ 1.2 and Q-value ≤ 0.05. The heatmap, Venn diagram, KEGG pathway, and GO analysis of DEGs were performed using the Dr. Tom Multi-omics Data Mining System (https://biosys.bgi.com), accessed on 25 October 2021.

### 4.13. Accession Numbers

We acquired all the Arabidopsis gene sequences from the TAIR database: *VGT1 (AT3G03090)*, *TMT1 (AT1G20840)*, *TMT2 (AT4G35300)*, *TMT3 (AT3G51490)*, *SAUR22 (AT5G18050)*, *SAUR23 (AT5G18060)*, *SAUR26 (AT3G03850)*, *SWEET2 (AT3G14770)*, *ERDL6 (AT1G75220)*, *DAO2 (AT1G14120)*, *PIN6 (AT1G77110)*, *CYP79B3 (AT2G22330)*, *TOR (AT1G50030)*, *KIN11 (AT3G29160)*, *E2F1 (AT5G22220)*, *ACTIN2 (AT3G18780)*, *ACTIN8 (AT1G49240)*.

## Data Availability

Raw RNA-seq data have been deposited in the Gene Expression Omnibus (GEO) database with the accession code GSE226876.

## Supplementary Materials

The following supporting information can be downloaded at: https://www.mdpi.com/article/10.3390/ijms242115852/s1.
