# Supplementary material for: Vacuolar Sugar Transporter TMT2 Plays Crucial Roles in Germination and Seedling Development in Arabidopsis
Source: Int J Mol Sci. 2023 Nov 1;24(21):15852. doi: 10.3390/ijms242115852 (PMC10647555; doi:10.3390/ijms242115852)
Supplement: Supplementary file 1 [file ijms-24-15852-s001.zip › ijms-2638948-supplementary.pdf]

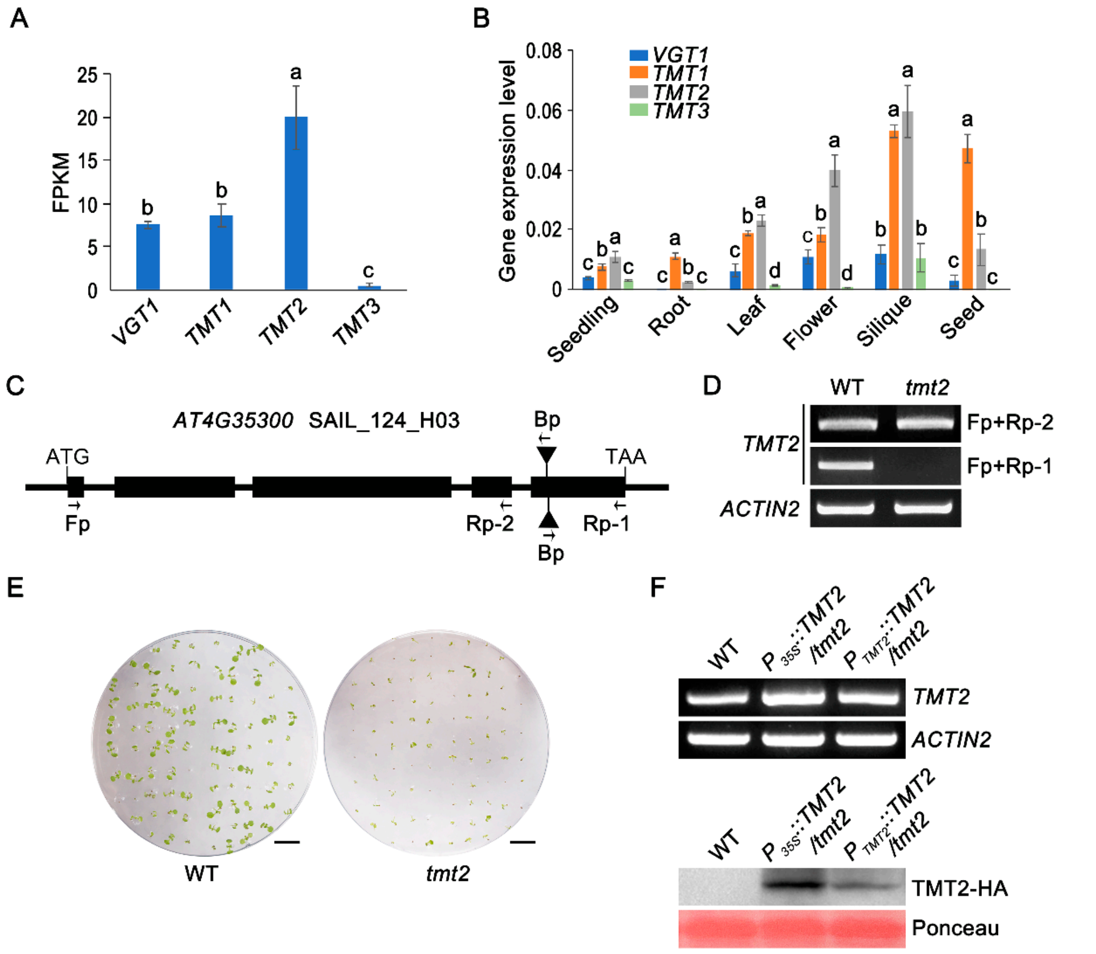

Supplemental Figure S1. Characterization of the *TMT2* gene and the *tmt2* mutant. (**A**) Gene expression levels of *VGT1*, *TMT1*, *TMT2*, and *TMT3* in 10-day-old wild-type (WT) seedlings achieved from RNA-seq analysis. FPKM: Fragments per kilobase of transcript per million mapped fragments. (**B**) Quantitative RT-PCR (qPT-PCR) analysis for the expression of *VGT1*, *TMT1*, *TMT2*, and *TMT3* in different tissues of WT Arabidopsis. *ACTIN8* was used as the reference gene. Error bars in **A** and **B** represent the standard deviation (SD) calculated from triplicate experiments. Different letters (a-d) indicate statistically significant differences among samples using one-way ANOVA with Tukey's mean test ($P < 0.05$). (**C**) The schematic diagram of the *TMT2* gene and its T-DNA insertion site (black triangle). The black arrows represent the primers used for RT-PCR analysis in **D**. (**D**) RT-PCR analysis of *TMT2* transcripts in WT and *tmt2* plants. *ACTIN2* was used as the reference gene. (**E**) Seed germination and seedling initial growth of 7-day-old WT and *tmt2* on 1/2 MS with the coagulant phytagel media. Three experiments were performed. Scale bars = 1 cm. (**F**) Expression analysis of *TMT2* at RNA and Protein levels in 7-day-old WT, P_{35S}::*TMT2*/*tmt2* and P_{TMT2}::*TMT2*/*tmt2* complementation lines. Up panel: Semi-quantitative RT-PCR analysis of *TMT2*. *ACTIN2* was used as the reference. Bottom panel: Immunoblot analysis of TMT2-HA encoded by transferred P_{35S}::*TMT2* and P_{TMT2}::*TMT2* with anti-HA polyclonal antibody. The ponceau staining shows the equal loading of protein samples.

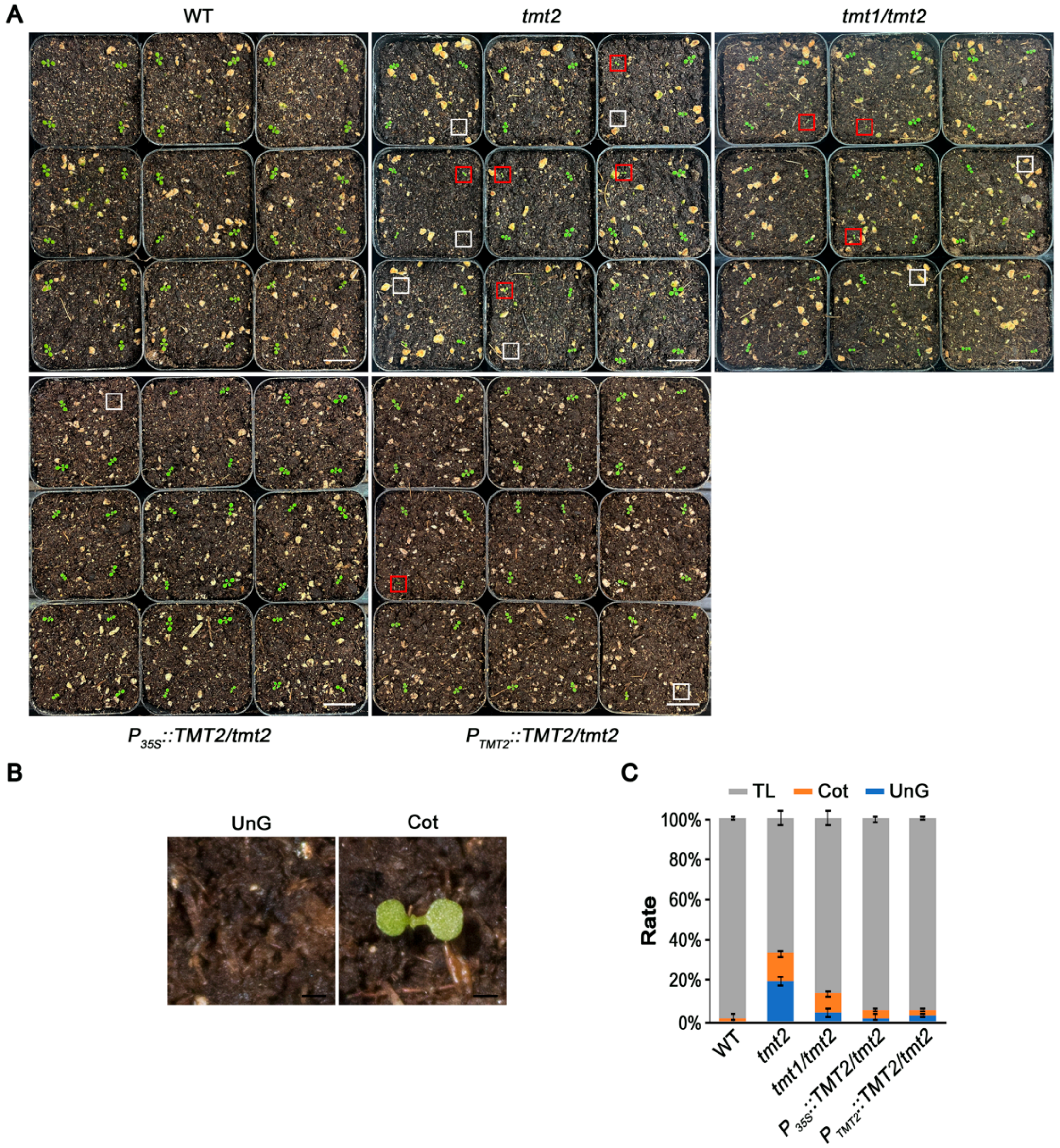

Supplemental Figure S2. Germination and initial growth of WT, *tmt2*, *tmt1*/*tmt2*, P_{35S}::*TMT2*/*tmt2* and P_{TMT2}::*TMT2*/*tmt2* complementation lines in the soil. (**A**) Growth of 10-day-old seedlings planted in the soil. Scale bars = 2 cm. White squares encompass un-germinated seeds (UnG), and red squares encompass seedlings arrested at the cotyledon stage (Cot). (**B**) Enlarged images of UnG and Cot in **A**. Scale bars = 1 mm. (**C**) Quantification results in **A**. A total of 252 seeds of each line were planted in the soil and growth was analyzed after 10 days. Error bars represent the SD of triplicate experiments.

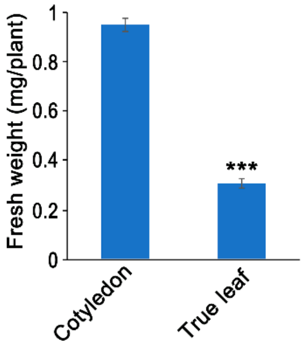

Supplemental Figure S3. Fresh weight of cotyledons and true leaves from 7-day-old WT seedlings on 1/2 MS media. Cotyledons and true leaves from 60 seedlings were analyzed. Error bars represent the SD of three biological replications. Significant differences are indicated as ***, P < 0.001 by the Student's t-test.

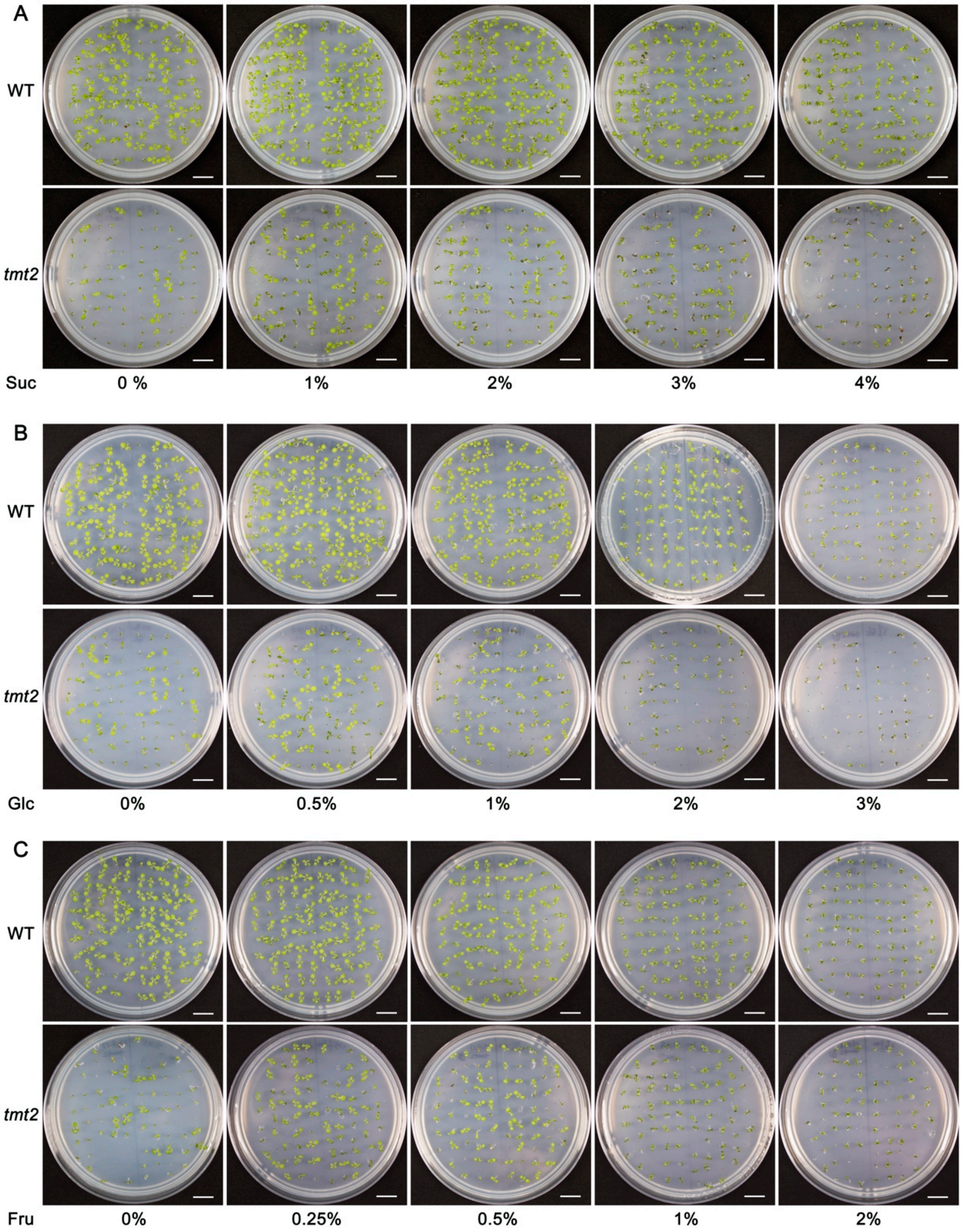

Supplemental Figure S4. Phenotypes of WT and *tmt2* seedlings on 1/2 MS media with different sugars. Initial growth of 7-day-old WT and *tmt2* seedlings on 1/2 MS media with different contents of sugars: (**A**) sucrose, (**B**) glucose, and (**C**) fructose. Each experiment is a representative of three repeats. Scale bars = 1 cm.

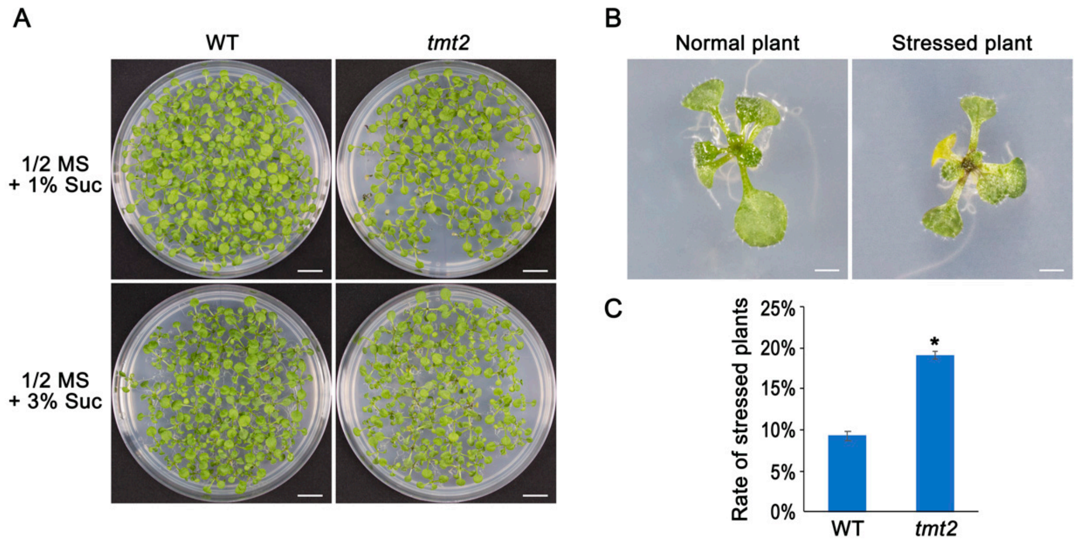

Supplemental Figure S5. Sensitivity of WT and *tmt2* plants to high sugar. (**A**) Top panel: WT and *tmt2* seedlings were grown for 12 days post-germination on 1/2 MS media with 1% sucrose. Bottom panel: WT and *tmt2-L* seedlings were initially grown for 5 days on 1/2 MS media with 1% sucrose and then were transferred onto 1/2 MS media with 3% sucrose for another 7 days of growth. Three experiments were repeated. Scale bars = 1 cm. (**B**) Enlarged images of normal and stressed plants on 1/2 MS media with 3% sucrose were presented. Plants with purple leaves were recorded as stressed plants. Scale bars = 1 mm. (**C**) Statistics analysis of stressed plants on 1/2 MS media with 3% sucrose in **A** was performed. A total of 234 seedlings of the WT and *tmt2-L* were analyzed. Error bars represent the SD of three biological replications. Significant differences are indicated as *, $P < 0.05$ by the Student's t-test.

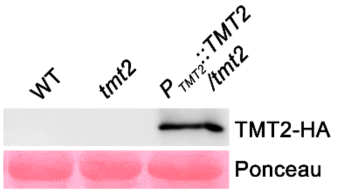

Supplemental Figure S6. The protein level of TMT2 in the cotyledons. The cotyledon samples were taken from 7-day-old WT, *tmt2*, and P_{TMT2}*::TMT2/tmt2* plants. The ponceau staining was used for the normalization of the TMT2 protein, which was immunoblotted against an anti-HA antibody.

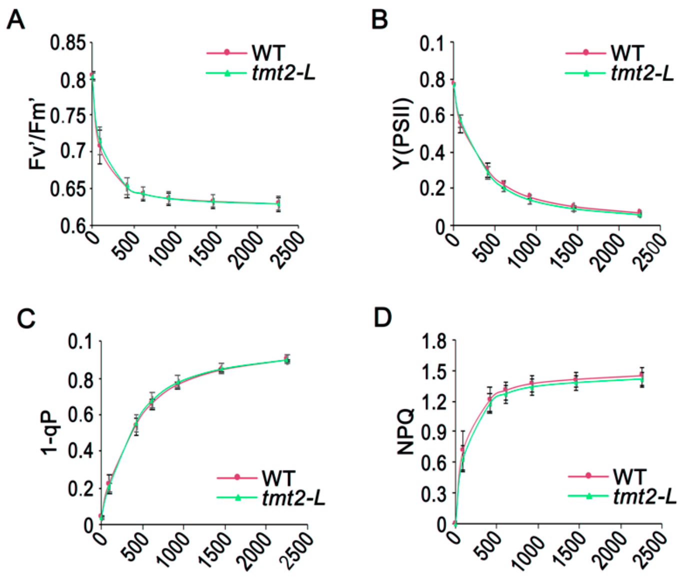

Supplemental Figure S7. Photosynthesis parameter analysis of WT and *tmt2-L* plants. (**A**) The light-induced curves of Fv′/Fm′, (**B**) Y(PSII), (**C**) 1−qP, and (**D**) NPQ. Chlorophyll fluorescence analysis was performed on leaves of WT and *tmt2-L* plants grown for 24 days in the soil. Statistical analysis was performed on six biological replicates using the Student's t-test.

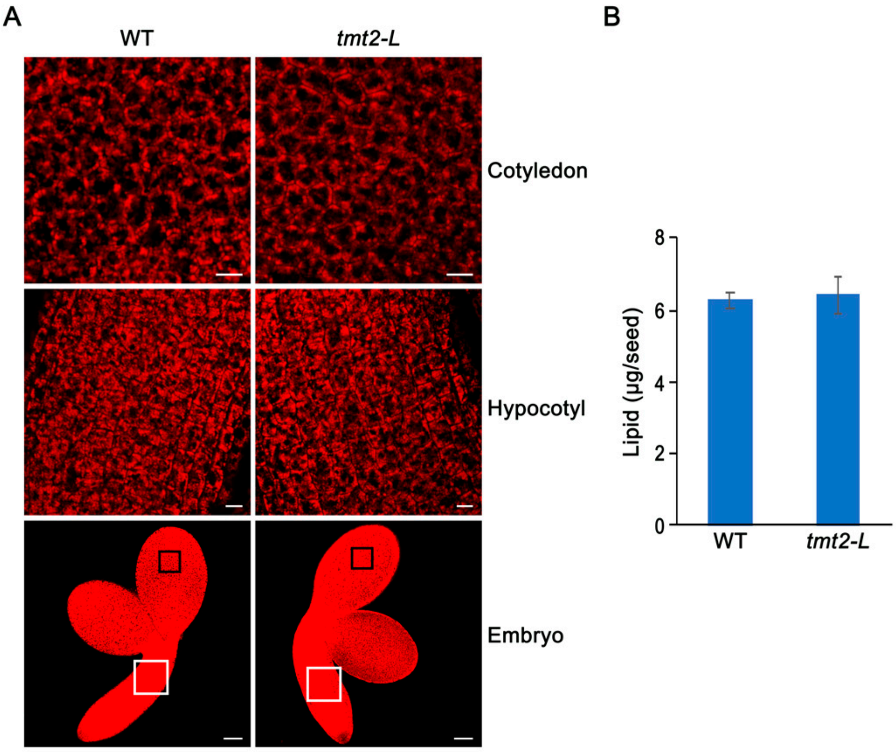

Supplemental Figure S8. Analysis of lipid droplets and lipid contents of WT and *tmt2-L* embryos and seeds. (**A**) Lipid droplets of WT and *tmt2-L* embryos at torpedo stage on the seventh day post-pollination stained with Nile red. Top: cotyledon, middle: hypocotyl, bottom: embryo. The black squares and white squares on embryos indicate the cotyledon and hypocotyl localization of image acquisition, respectively. Scale bars = 10 μm. (**B**) Lipid contents of WT and *tmt2-L* seeds. Error bars represent the SD of three biological replicates.

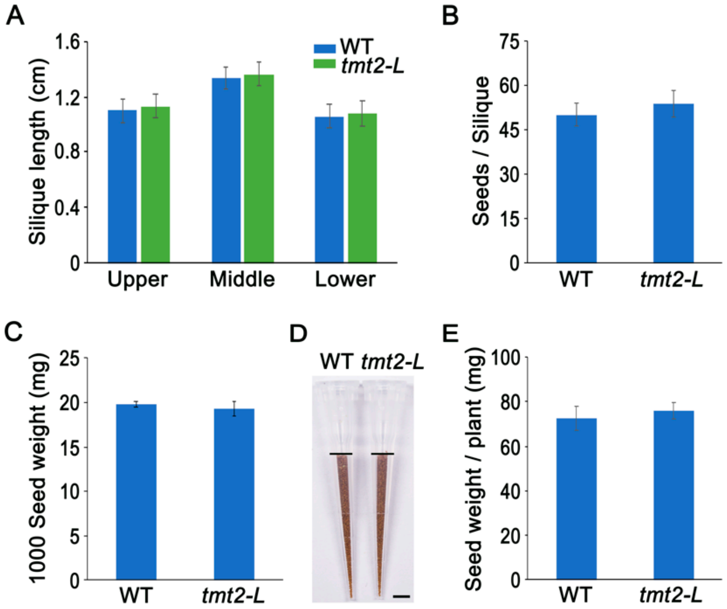

Supplemental Figure S9. Silique and seed analysis of WT and *tmt2-L* plants. (**A**) Length of siliques from the upper, middle, and lower parts of WT and *tmt2-L* plants. A total of 60 siliques from 12 plants were sampled. (**B**) Number of WT and *tmt2-L* seeds per silique. A total of 60 siliques from 12 plants were counted. (**C**) Weight of 1000 seeds from WT and *tmt2-L* plants with 3 independent pools. (**D**) 1000 seeds of WT and *tmt2-L* plants in tips. Scale bars = 0.5 cm. (**E**) Seed weights of WT and *tmt2-L* per plant. A total of 12 plants were sampled. Error bars represent the SD of triple repeats. Statistical analysis was performed between WT and *tmt2-L* plants using the Student's t-test.

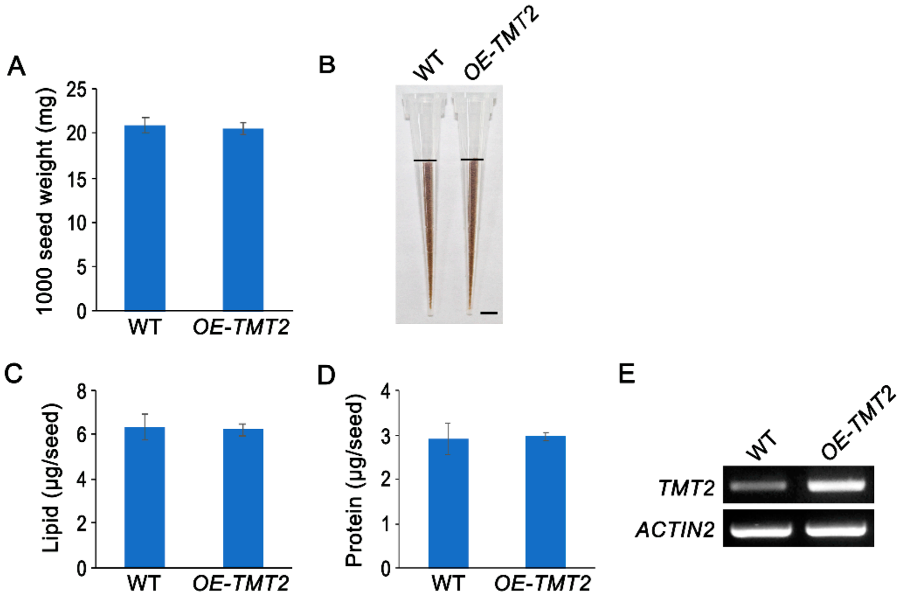

Supplemental Figure S10. Silique and seed analysis of WT and *OE-TMT2* plants. (**A**) The weight of 1000 seeds from WT and *OE-TMT2* plants with 3 independent pools. (**B**) The volume of 1000 seeds from WT and *OE-TMT2* plants. Scale bars = 0.5 cm. (**C**) Lipid contents of WT and *OE-TMT2* seeds. (**D**) Protein contents of the WT and *OE-TMT2* seeds. Statistical analyses in **A**, **C**, and **D** were performed using the Student's t-test. (**E**) RT-PCR analysis of *TMT2* transcripts in 3-week-old WT and *OE-TMT2* plants. The *OE-TMT2* overexpression transgenic line was generated by transforming P_{35S}::*TMT2* into WT plants. The transcript of *ACTIN2* was used as the reference.

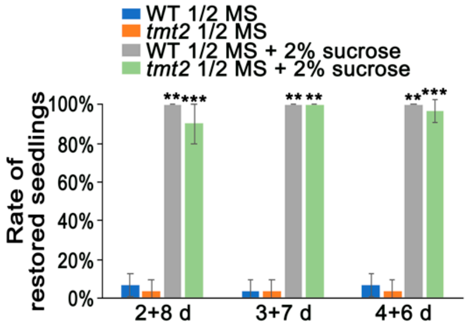

Supplemental Figure S11. Rates of normal seedlings of cotyledon-defoliated WT and *tmt2* plants grown on 1/2 MS media with or without 2% sucrose. WT and *tmt2* seedlings were initially grown on 1/2 MS media, and their cotyledons were removed on the second, third, or fourth day post-germination. The cotyledon-defoliated plants were further transferred onto 1/2 MS media without sucrose or 1/2 MS media with 2% sucrose for further growth until the tenth day post-germination. A total of 30 plants were analyzed. Error bars represent the SD of three biological replicates. Significant differences are indicated as **, $P < 0.01$ and ***, $P < 0.001$ by the Student's t-test.

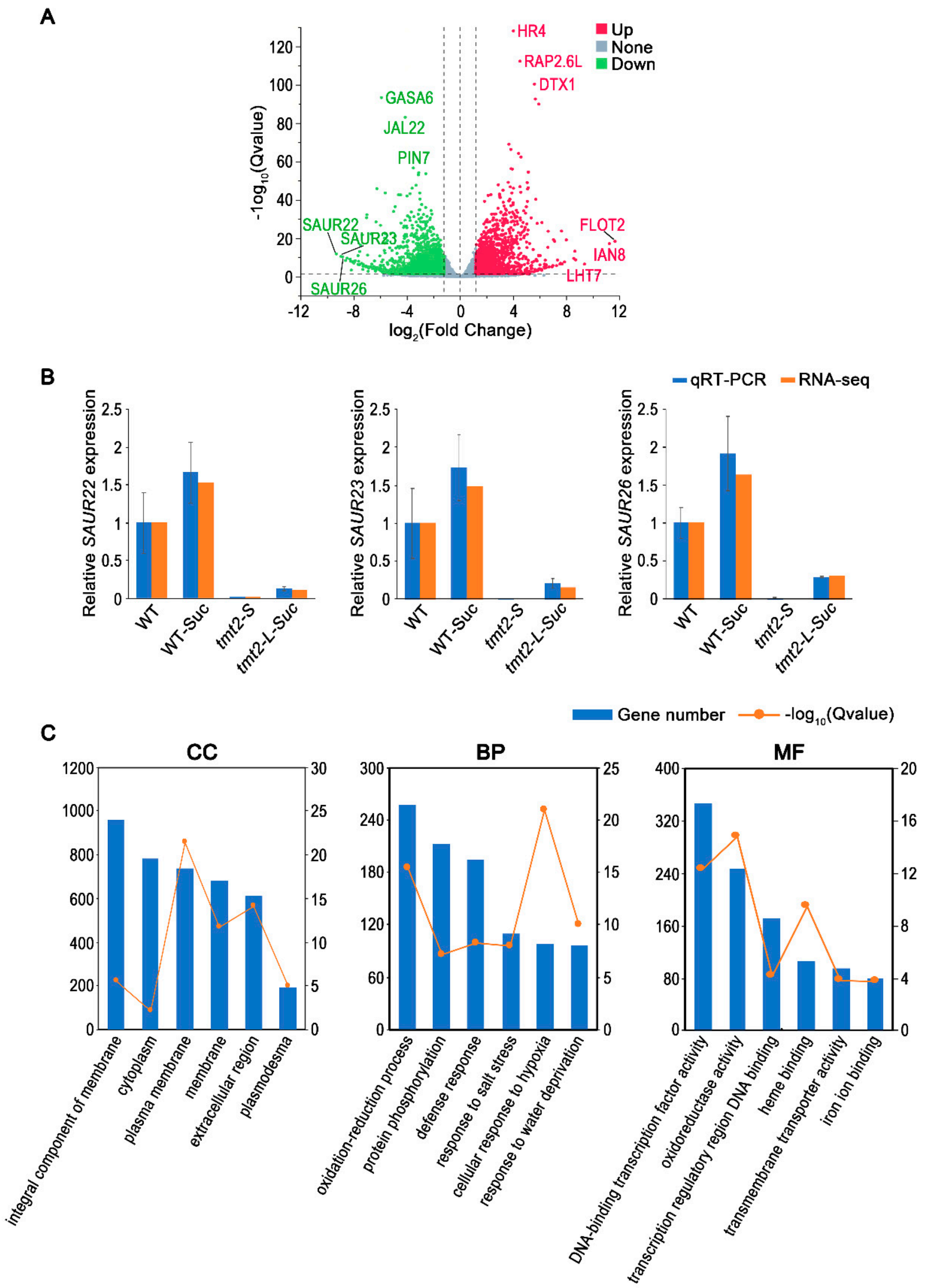

Supplemental Figure S12. Volcano map and GO category of *tmt2* / WT DEGs and validation of RNA-seq data by qRT-PCR. (**A**) Volcano map of *tmt2-S* / WT DEGs. The red color represents up-regulated DEGs, the blue color represents down-regulated DEGs, and the grey color represents non-DEGs. (**B**) The expression levels of *SAUR22*, *SAUR23*, and *SAUR26* in the WT, WT-Suc, *tmt2-S*, and *tmt2-L-Suc* seedlings validated by qRT-PCR in comparison to the RNA-seq data. *ACTIN8* was used as the reference gene. Error bars represent the SD of triple repeats. (**C**) GO functional enrichment analysis of *tmt2* / WT DEGs.

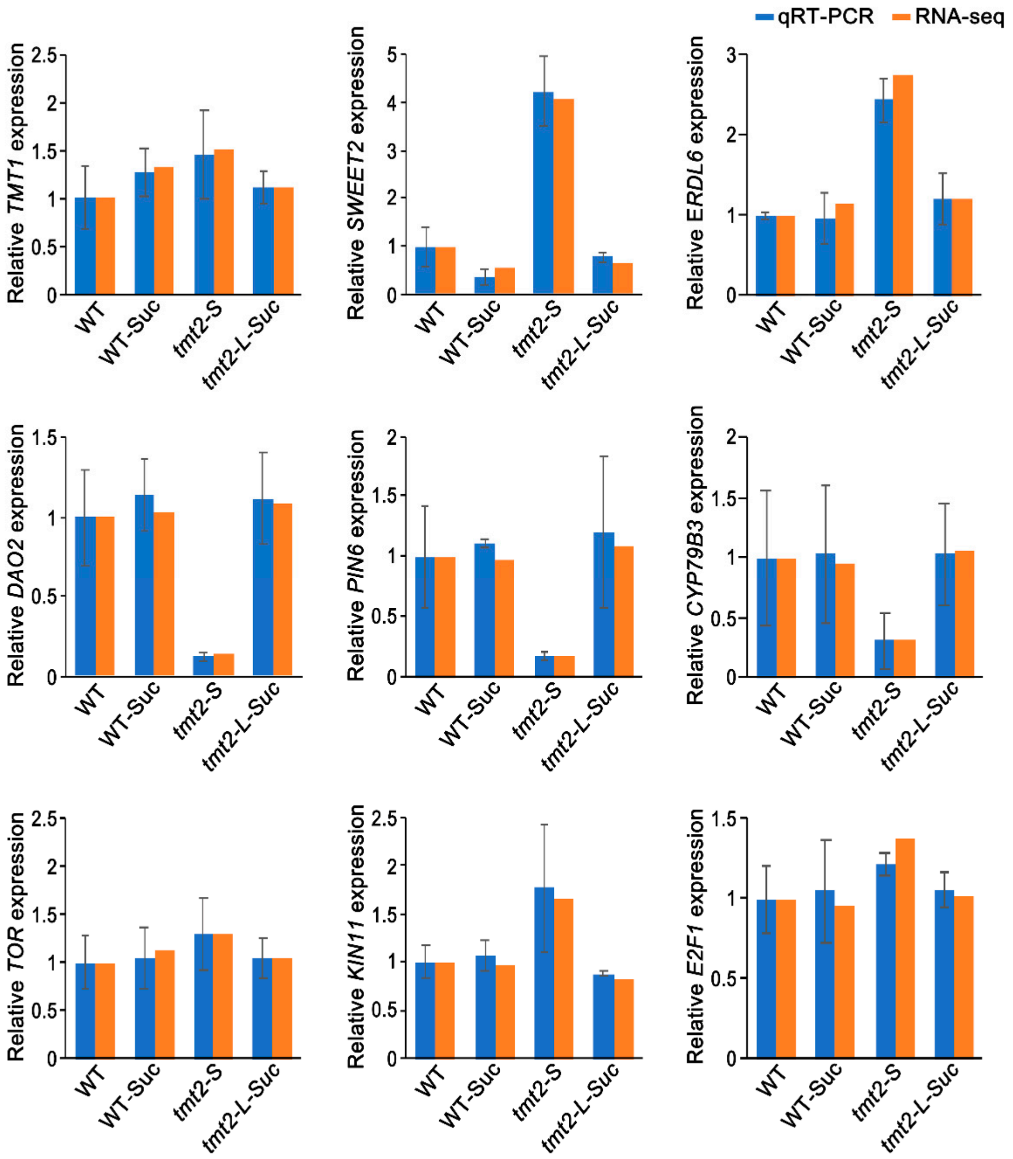

Supplemental Figure S13. Expression levels of representative genes of *tmt2* / WT DEGs retested by qRT-PCR. The expression levels of *TMT1*, *SWEET2*, *ERDL6*, *DAO2*, *PIN6*, *CYP79B3*, *TOR*, *KIN11*, and *E2F1* in the WT, WT-Suc, *tmt2-S*, and *tmt2-L-Suc* were validated by qRT-PCR in comparison to the RNA-seq data. *ACTIN8* was used as the reference gene. Error bars represent the SD of triple repeats.

Supplemental Table S1: List of primers used in this study

Primer name	Primer sequences (5'-3')	Function
VGT1-Fp-qRT	CCGGAATTCATGGGGTTTGATCCCGAGAAC	qRT-PCR analysis of *VGT1* gene
VGT1-Rp-qRT	TGCGTCGACCGGAGGAATGGCTGCAAGAAC	
TMT1-Fp-qRT	CGGAGAGATTTCAAGGCAAAGCT	qRT-PCR analysis of *TMT1* gene
TMT1-Rp-qRT	GTCTGCCGAGCCAATCAGATATC	
TMT2-Fp-qRT	GTCGAATTCATGAGTGGAGCTGTGCTTGTTGC	qRT-PCR analysis of *TMT2* gene
TMT2-Rp-qRT	CATGTTGTAATCAGAGTAGCACC	
TMT3-Fp-qRT	ATGAGGAGTGTAGTGCTTGTTGC	qRT-PCR analysis of *TMT3* gene
TMT3-Rp-qRT	AAGTGGCTCCAATGAGAGACAT	
tmt2-Lp	AGTTCTTCCTCAGCATCCTTACT	Identification of *tmt2* mutant
tmt2-Rp-1	GTCGTCGACTCACTCGTTTTTAGCAGCTTCAGC	
SAIL-Bp	GCCTTTTCAGAAATGGATAAATAGCCTTGCTTCC	
tmt2-Fp	GTCGAATTCATGAGTGGAGCTGTGCTTGTTGC	RT-PCR analysis of *tmt2* mutant
tmt2-Rp-1	GTCGTCGACTCACTCGTTTTTAGCAGCTTCAGC	
tmt2-Rp-2	CTTCGACCAGAAAGATCCATGAGC	
tmt2-Lp	AGTTCTTCCTCAGCATCCTTACT	RT-PCR analysis of P_{35S}::*TMT2*/*tmt2* and P_{TMT2}::*TMT2*/*tmt2* lines
tmt2-Rp-1	GTCGTCGACTCACTCGTTTTTAGCAGCTTCAGC	
TMT2-Fp (pRI101-AN)	ATAGTCGACATGAGTGGAGCTGTGCTTGTTGC	Constructs of pRI101-P_{35S}::*TMT2* -*HA* and pRI101-P_{TMT2}::*TMT2-HA*
TMT2-HA-Rp (pRI101-AN)	GCCGAATTCTCAAGCGTAATCTGGAACATC<br>GTATGGGTACTCGTTTTTAGCAGCTTCAG	
35S-F	ATGACGCACAATCCCACTATC	Identification of transgene plants with *TMT2* gene
tmt2-Rp-1	GTCGTCGACTCACTCGTTTTTAGCAGCTTCAGC	
ACTIN2-Fp	CTCTCCCGCTATGTATGTCGCCAT	RT-PCR analysis of *ACTIN2* gene
ACTIN2-Rp	TGTGAACGATTCCTGGACCTGCCT	
P_{TMT2}-Fp (pCAMBIA1305)	GATCTGCAGCTGTGGTGTGTACTGTAGTTCC	Constructs of pCAMBIA1305-P_{TMT2}::*GUS* and
P_{TMT2}-Rp (pCAMBIA1305)	GCGCCATGGAATTCCGTCTTCTATCTTTTATC	

		pRI101-*P_{TMT2}*:: *TMT2-HA*
GUS-Fp	CGTATCGTGCTCCGCTTCGGCTCTG	Identification transgene plants with *P$_{TMT2}$-GUS* gene
GUS-Rp	TCTTCATAGACATCGATGGTCAGTC	
TMT2-Fp (pCAMBIA2300)	ATCGGTACCATGAGTGGAGCTGTGCTTGTTGC	Construct of pCAMBIA2300-*P$_{35S}$::TMT2-eGFP*
TMT2-Rp (pCAMBIA2300)	GCAGTCGACCTCGTTTTTAGCAGCTTCAGCTTG	
eGFP-Fp	GTGAAGGTGATGCAACATACGGAA	Identification of transgene plants with *TMT2-eGFP* fused gene
eGFP-Rp	GTGGACAGGTAATGGTTGTCTGGT	
SAUR22-Fp-qRT	ATGGCTCTGGTGAGAAGTCTACT	qRT-PCR analysis of *SAUR22* gene
SAUR22-Rp-qRT	TTGGTTCAAGTATGAGAGTGGCA	
SAUR23-Fp-qRT	ATGGCTTTGGTGAGAAGTCTAT	qRT-PCR analysis of *SAUR23* gene
SAUR23-Rp-qRT	GCTGGTTCAAGTATGAGAGTGGC	
SAUR26-Fp-qRT	ATGGCTTTGGTGAGAAGTCTCTT	qRT-PCR analysis of *SAUR26* gene
SAUR26-Rp-qRT	GGTTCAAGTATGAGACTGGTACAA	
ACTIN8-Fp	GGTTCTACTTACCGAGGCTCC	qRT-PCR analysis of *ACTIN8* gene
ACTIN8-Rp	GATAGGCACAGTGTGAGACAC	
